# Delay discounting in adolescence depends on whom you wait for: Evidence from a functional neuroimaging study

**DOI:** 10.1016/j.dcn.2024.101463

**Published:** 2024-10-18

**Authors:** Lotte H. van Rijn, Suzanne van de Groep, Michelle Achterberg, Lara Wierenga, Barbara R. Braams, Valeria Gazzola, Berna Güroğlu, Christian Keysers, Lucres Nauta-Jansen, Anna van Duijvenvoorde, Lydia Krabbendam, Eveline A. Crone

**Affiliations:** a Department of Psychology, Education and Family Studies, Erasmus School of Social and Behavioral Sciences, Erasmus University Rotterdam, the Netherlands; b Leiden University, Institute of Psychology, Developmental and Educational Psychology Department, Leiden, the Netherlands; c Department of Clinical, Neuro, and Developmental Psychology, Faculty of Behavioral and Movement Sciences, Vrije Universiteit Amsterdam, the Netherlands; d Social Brain Lab, Netherlands Institute for Neuroscience (KNAW), the Netherlands; e University of Amsterdam, Amsterdam, the Netherlands; f Department of Child and Adolescent Psychiatry & Psychosocial Care, AmsterdamUMC, location Vrije Universiteit Amsterdam and Research Institute Amsterdam Public Health, Amsterdam, the Netherlands

**Keywords:** Delay discounting, FMRI, social, Self-regulation, Adolescence, Reward, Friend, Unknown other

## Abstract

With age, adolescents increasingly demonstrate the ability to forgo immediate, smaller rewards in favor of larger delayed rewards, indicating reduced delay discounting. Adolescence is also a time of social reorientation, where decisions not only involve weighing immediate against future outcomes, but also consequences for self versus those for others. In this functional Magnetic Resonance Imaging study, we examined the neural correlates of immediate and delayed reward choices where the delayed outcomes could benefit self, friends, or unknown others. A total of 196 adolescent twins aged 14–17 completed a social delay discounting task, with fMRI data acquired from 174 participants. Out of these, 156 adolescents had valid fMRI data, and 138 adolescents had observations in every condition. Adolescents more often chose the immediate reward when it was larger, and when the delay was longer. Area-under-the-curve (AUC) comparisons revealed that behavior differed across delay-beneficiaries, with AUC being highest for the self, followed by friends, and lowest for unknown others. This suggests that adolescents are more willing to wait for rewards for self. Neuroimaging analyses showed increased activity in the midline areas medial prefrontal cortex (MPFC) and precuneus, as well as bilateral temporal parietal junction (TPJ) when considering delayed reward for unknown others and friends compared to self. A whole-brain interaction with choice showed that the bilateral insula and right dorsolateral prefrontal cortex (DLPFC) were more active for delayed choices for unknown others and for immediate choices for friends and self. This underscores that the neuro-cognitive processing of how delays reduce the value of rewards depends on closeness of the beneficiary.

## 1 Introduction

Many of our daily decisions involve balancing short-term and long-term benefits. Prior studies have shown that when individuals choose between an immediate smaller reward and a larger delayed reward, individuals are less willing to wait for the delayed reward when this reward is further in the future, a process also known as delay discounting (Luerssen et al., 2015, Olson et al., 2009). Across childhood and adolescence, the willingness to wait for a larger reward increases with age, suggesting that adolescents develop the cognitive resources to increasingly invest in future outcomes and overcome the tendency to choose for an immediate reward (Achterberg et al., 2016, Steinberg et al., 2008, van den Bos et al., 2014). Possibly, these developments are associated with an increased ability to think of oneself in the future and value the needs one may have in the future (Pfeifer and Peake, 2012, Van den Bos et al., 2016), to control impulses to choose for the immediate reward (Steinberg et al., 2008), and to maximize overall outcomes by waiting for the larger reward (Peper et al., 2018).

However, many of our decisions are not solely driven by temporal considerations, but also by the balance between outcomes for self and others (Crone and Fuligni, 2020). Specifically, during adolescence there is a shift from thinking about oneself across temporal dimensions towards thinking about oneself across social dimensions (Nelson et al., 2016). Indeed, prior research has shown that adolescents’ delay discounting is influenced by whether immediate and delayed outcomes benefit themselves or a friend, such that they prefer immediate rewards for themselves when juxtaposed to a delayed reward for a friend (van de Groep et al., 2023). These target differentiations are also observed outside of the circle of close friends. A set of recent studies has demonstrated that sharing resources between self and others depends on the closeness of the relationship, and that adolescents increasingly differentiate between sharing targets during adolescence (Guroglu et al., 2014). Specifically, sharing resources with friends and family members increases with age, whereas sharing with unknown others decreases with age (Karan et al., 2022, van de Groep et al., 2020). This suggests a possible emergence of an increased ingroup preference during adolescence (Guassi Moreira et al., 2017, Hughes et al., 2017). Balancing outcomes for self and others could thus be influenced by the relationship with the other, where decisions might differ when friends or more distant, societal partners are involved (Crone et al., 2024). A better understanding of decision making that involves more distant others can inform the degree to which personal connection affects the valuation of delayed rewards. This will contribute to our understanding of how adolescents make decisions with broader, long-term benefits beyond immediate personal benefit, and thus how adolescents navigate social and societal goals in an increasingly complex society.

Several studies have examined the neural correlates of choosing for self and others in the present and in the future, and these studies have unraveled separable brain networks when making delay discounting choices that benefit self or friends (Albrecht et al., 2011, van de Groep et al., 2023). Opting for delayed rewards for oneself is associated with increased activity in cognitive control regions of the brain, including the dorsolateral prefrontal cortex and parietal cortex (Christakou et al., 2011, McClure et al., 2004). In contrast, making delayed choices for friends is associated with increased activity in the precuneus (Albrecht et al., 2011, van de Groep et al., 2023) and temporal parietal junction (van de Groep et al., 2023), regions integral to the social brain network primarily engaged in mentalizing (Burnett et al., 2011). The functional role of the medial prefrontal cortex remains somewhat ambiguous. Some studies identify it as a key area for thinking about oneself across time (past, present and future) and therefore it is likely to be involved in future orientation (D'Argembeau, 2013, Pfeifer and Peake, 2012, van den Bos et al., 2015). Conversely, other studies emphasize its importance for thinking about intentions and consequences for others as part of the social brain network, or specifically the mentalizing network (Amodio and Frith, 2006).

Taken together, recent studies have demonstrated that during adolescence, there is social reorientation towards outcomes that benefit friends (van de Groep et al., 2020) but less so for outcomes that benefit unknown others (Karan et al., 2022, van de Groep et al., 2020), and delay choices for friends are associated with activation in a separable set of brain regions compared to making choices for self (van de Groep et al., 2023). However, the neural correlates for delay decisions for more distant others remain unknown, even though social reorientation during adolescence requires individuals to make connections with people outside their close circle of friends and family (Fuligni, 2019, Telzer, 2016), for example when moving to campus or when connecting to new, unfamiliar classmates. Besides, a society benefits from a better understanding of what drives adolescents to overcome immediate benefits for self to benefit unknown others in the future, such as is the case with volunteering, fundraising, vaccinations, adhering to rules and group projects.

A recent fMRI study demonstrated that sharing with unknown others, relative to known others, resulted in increased activity in the dorsal anterior cingulate cortex and bilateral insula, which was interpreted to suggest that these decisions reflect a deviation of personal norms (Schreuders et al., 2018) or increased saliency (Rilling and Sanfey, 2011). However, the specifics of how making delay choices for distant others is associated with activation in these brain regions remain unknown, providing an avenue for further exploration.

The goal of this explorative study was to test the behavioral patterns and neural correlates of delay discounting choices for targets that differ in personal closeness in mid adolescence. For this purpose, we invited adolescents between ages 14–17-years to perform a delay discounting task where smaller immediate rewards for self were pitted against larger delayed rewards for self, friends, and unknown others. Participants were monozygotic and dizygotic twins that were included in in the Leiden-Consortium on Individual Development study (Crone et al., 2020) which provided us with the unique possibility to exploratively test whether delay discounting choices are influenced by genetic, shared environment, or unique environments when growing up (Achterberg et al., 2018). All choices were made relative to immediate self-outcomes to test whether participants were willing to sacrifice an immediate self-benefit for larger benefits in the future for themselves, their friends, and unknown others.

We hypothesized that during choices for an immediate smaller reward for self relative to a larger delayed reward for self, participants would show increased activity in the ventral striatum and subgenual ACC (van de Groep et al., 2023), where we expected increased activity in the lateral prefrontal cortex, dorsal medial prefrontal cortex, and parietal cortex for delayed outcomes (van den Bos et al., 2015). Despite the relatively narrow age range, we expected an age-related decrease in delay discounting (Peper et al., 2018, van den Bos et al., 2015).

Additionally, delay discounting was expected to be steeper for unknown others than for friends and self. Thus, with increasing delay, we expected individuals to choose the immediate reward increasingly more when the delay-beneficiary was unknown other compared to friend or self. We hypothesized that making choices for friends and unknown others would be associated with increased activity in the mentalizing network, including the dorsal medial prefrontal cortex, precuneus, and temporal parietal junction (van de Groep et al., 2023) and we explored whether this activity was different for close (i.e., friends) versus distant (i.e., unknown others) targets. Finally, we hypothesized an interaction between type of choice (i.e., immediate versus delayed) and the beneficiary (self, friend, unknown others) in these networks (Schreuders et al., 2018), where we expected that the delayed choices would lead to stronger activity for distant others. Based on prior studies that demonstrated individual differences in brain activity depending on the individual differences in delay discounting behavior, we also expected that activity in the cognitive and social brain regions would correlate with behavioral delay discounting scores (van de Groep et al., 2023, van den Bos et al., 2015).

## 2 Method

### 2.1 Participants

Participants were recruited from the middle childhood cohort of the Leiden Consortium on Individual Development (L- CID), which is a sequential longitudinal twin study (Crone et al., 2020). Twins were initially recruited through municipality records in the Netherlands and were eligible if both parents were fluent in Dutch and both their parents and grandparents originated from Europe (Euser et al., 2016). Children were initially excluded from participation if they reported a congenital disability, psychological disorder, chronic illness, hereditary disease, hearing impairment, or visual impairment that could influence their behavioral performance. Additionally, if children were diagnosed with an intellectual disability at the time of initial inclusion, (IQ < 70) they were also excluded from participation.

In total, we included 196 adolescents (IQ at initial inclusion available for *N* = 194, *M* = 103.74, *SD* = 11.46, range = 75–137.5) of which 174 adolescents performed the task inside the scanner (48.3 % females, *M*_age_ = 15.75, *SD*_age_ =.80, age range = 14.25 – 17.22) and 22 participants performed the task outside of the scanner (45,5 % females, *M*_age_ = 15.27, *SD*_age_ =.77, age range = 14.25 – 16.96). All 196 participants were included in the behavioral analyses. For the MRI analysis, five participants were excluded due to technical loss (parts of the masks missing, or incomplete data due to technical issues with the MRI scanner). Another 13 participants were excluded because of excessive movement, which was determined with a cutoff of > 3 mm for the maximum movement over all directions (x, y, z) per run. If excessive movement was observed in one or both runs, participants were excluded, resulting in a sample of 156 subjects for the MRI analyses (48.8 % females, *M*_age_ = 15.78, *SD*_age_ =.77, age range = 14.27 – 17.14) (Figure A.1). This method was chosen as some choice conditions are based on few trials, and this method allowed to optimize the number of trials for the MRI analysis (see also Figure C.1).

The study was approved by the Dutch Central committee on Research Involving Human Subjects (CCMO).

### 2.2 Materials: social delay discounting task

Participants performed a modified version of the Social Delay Discounting Task (van de Groep et al., 2023). Participants played the task for themselves, a friend, and an unknown other. Participants made choices in three experimental condition each with a different delay-beneficiary: 1) a small (2–8€) reward for the self now, or a 10€ reward for the self later (‘self now – self later’ condition), 2) a small (2–8€) reward for the self now, or a 10€ reward for a friend later (‘self now – friend later’ condition), 3) a small (2–8€) reward for the self now, or a 10€ reward for an unknown other later (‘self now – unknown other later’ condition). Additionally, a control condition was included in which participants had to select the largest of two circles (van de Groep et al., 2023, de Water et al., 2017).

**Fig. 1:**
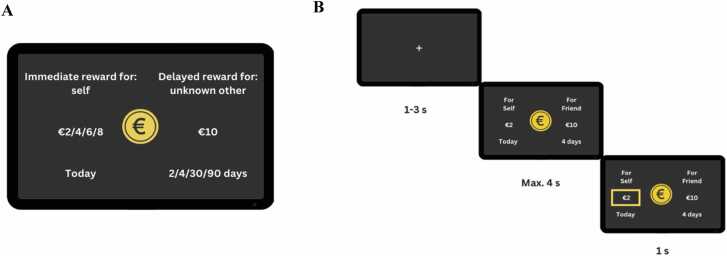
*Task design*. *Note.* (A) Visual display of the Social Delay Discounting Task. The task consisted of four conditions where participants had to choose between two options: 1) an immediate reward for the self or a delayed reward for the self, 2) an immediate reward for the self or a delayed reward for the friend, 3) an immediate reward for the self or a delayed reward for an unknown other. The fourth condition was a control condition. Both immediate rewards (2, 4, 6, 8) and the number of days as delay (2, 4, 30, 90) varied across trials. (B) Example trial of the Social Delay Discounting task. First a fixation screen was presented with a jittered duration between 1 and 3 seconds. Then the choice was presented for a maximum of 4 seconds, where participants could indicate the left choice with their right index finger, or the right choice with their right middle finger. A confirmation screen was then presented for 1 second, where the choice was highlighted with a yellow frame.

For each experimental trial both the immediate reward magnitude (2, 4, 6, 8€) and the delay in days (2, 4, 30, 90 days) varied (Fig. 1A). The delayed reward was always 10€. The original task additionally included a delay of 0 for the delay-beneficiaries friend and unknown other, but these were not included in the current analysis as this was not comparable over all groups (i.e., with the self as delay-beneficiary). To keep trial numbers consistent over conditions, additional trials were added in the self-condition for immediate reward 4 and 6 to compensate for the additional trials with a delay of 0 in the friend and unknown other condition. This choice was made as these reward values are often tipping indifference points where the costs and benefits compared to a delayed reward of 10 euros are weighted equally.

The task was presented using E-prime version 3 (Schneider et al., 2002; version 3.0). Every trial started with a jittered fixation cross ranging between 1000 ms and 3000 ms (Fig. 1B). The choice was then presented, and participants had 4000 ms to indicate their response using their right index finger (left option) or right middle finger (right option). After their decision, their choice was highlighted for 1000 ms via a yellow frame around the selected choice. If a choice was not made in time, a ‘too late’ screen was shown for 1000 ms and the trial was excluded from analysis. For the control trials the location of the circles (the largest one either left or right) was counterbalanced across blocks. Every experimental condition (‘self now – self later’, ‘self now – friend later’, ‘self now – unknown other later’) consisted of 40 trials in total that were divided over two runs by implementing a mini block (20 trials) of each condition in each run. The control condition consisted of 12 trials in total (2 trials in each mini block, thus 6 per run). The total duration of the task in the scanner was 15.4 min. See Table A.1 for an overview of the experimental trials. For the experimental trials, the immediate choice was always presented on the left, and the delayed choice was always presented on the right.

We randomly selected one trial for pay-out. Given that each choice could result in a pay-out, each choice was made as an immediate-delay choice, but choices were not dependent on prior trials, thereby removing the possibility of strategic behavior (e.g., immediate choices for half of the trials, delayed choices for the other half of the trials). Payments could either be allocated to the participant or a friend (whose identity was disclosed by the participant), while in this study we did not allocate payments to the unknown other (represented by a random name, either provided by the experimenter or the participant). Participants were not specifically informed that payment could not be allocated to the unknown other. Since a random trial was selected, payment could be either 2, 4, 6, 8 or 10 euros.

### 2.3 Procedure

Participants were contacted by telephone to assess their willingness to participate in the 7th wave of the L-CID study. Informed consent was provided at the start of the lab visit, where additional consent from parents was required for participants aged 15 or younger. As the current study was part of the 7th wave of data collection in a longitudinal study, participants completed additional tasks and questionnaires besides the Social Delay Discounting Task in the fMRI scan. Tasks and questionnaires were alternated with the fMRI scan, and the order was randomized over participants.

At the start of the lab visit participants were informed about the visit, the MRI scanner, and the other measurements. The MRI session was the same for each participant, consisting of a structural scan, two fMRI tasks (of which the second was the Social Delay Discounting Task), a resting state scan and a diffusion tensor imaging scan in corresponding order. Participants received 75 euros for their participation, with an additional payment based on the Social Delay Discounting Task in the MRI. The additional payment was paid along with the reimbursement for participation. If a trial was selected in which the participant had chosen the delayed reward for the friend, the participant was asked to give the money to the friend.

### 2.4 (f)MRI data acquisition

MRI scans were acquired on a Philips Achieva 3.0 T MRI system with a standard whole-head coil at the Leiden University Medical Centre. During the scanning session, head motion was limited with the use of foam inserts around the children’s heads, and stimuli were presented on a screen which was visible through a mirror attached to the head coil.

For anatomical reference, high resolution T1 weighted images were acquired (FOV) = 224 (ap) x 177 (rl) x 168 (fh); flip angle (FA) = 8º; voxel size = 0.875 ×0.875 ×0.875 mm). The duration of the T1- weighted scan was 296 seconds. Scan quality was checked for motion artifacts during the scan, where scans were repeated if motion was detected.

Functional scans were acquired during 2 runs, which were manually stopped when the pause screen appeared between the big blocks. The dynamics therefore differed per person. We collected T2 * weighted gradient echo planar images (EPI; TR = 2.2 s, TE= 30 ms, flip angle 80º, sequential acquisition: 37 slices, voxel size = 2.75 ×2.75 ×2.75 mm, 80 ×77 matrix, field of view (FOV) = 220 × 220 ×115 mm). Before the start of each functional run, 5 dummy scans were made. All scans were acquired using a fast field echo pulse sequence.

After excluding participants with excessive head motion (> 3 mm of maximum movement in any direction in one or both runs, *N* = 13), movement was as follows: range: .00 - 2.86 mm, *M* = 0.10, *SD* = 0.07 (see Table A.2, A.3 and A.4).

### 2.5 Behavioral analyses

We first calculated the Subjective Value (SV) for each delay (2, 4, 30, 90) per individual using the method by de Water et al. (2017); (2023). This can be calculated by summing the delay choices per delay and dividing them by the total number of choices per delay. This is then multiplied by the range of possible SVs in the task (8), after the lowest possible SV is added (1). See Appendix A for the exact calculation. Although one might initially think that the SVs can range from 0 to 10 (the absolute value), an SV of 0 assumes that the reward can completely lose its value, while an SV of 10 assumes a delay could have no impact on the reward’s value. Since this is not theoretically probable, the range is adjusted to 1–9, hence the range of possible SVs (8; de Water et al., 2023). The SVs were then divided by 10 to calculate normalized SVs, ranging from 0.1 to 0.9. The same method was applied for each immediate reward to get a measure of delayed choices for each immediate reward (2, 4, 6, 8). We then calculated a measure of delay discounting using Area Under the Curve (AUC) as described by Myerson et al. (2001). To calculate the AUC values, we used normalized delays as a proportion of the maximum delay. See Appendix A for the exact calculation. This procedure results in AUC values ranging from 0 to 1 and is also described in Myerson et al. (2001). A smaller value is associated with more immediate choices (de Water et al., 2023). We chose to apply this method to stay consistent with previous studies examining delay discounting in adolescence, and to allow for a measure of delay discounting that does not assume a specific shape of discounting function given the exploratory nature of our study examining delay discounting not only for self but also others (van de Groep et al., 2023, de Water et al., 2017).

We then conducted manipulation checks regarding participants’ certainty about the payments. This was assessed in an exit questionnaire that contained the question ‘Are you worried whether you will actually receive the money?’ which was rated on a scale from 1 to 10. This allowed us to see whether uncertainty influenced behavior in the task, by calculating the correlation between uncertainty and the AUC values for each delay-beneficiary. We additionally checked whether participants differentiated between delay-beneficiaries. As part of the exit questionnaire, participants rated closeness of the indicated friend and unknown other on the Inclusion of Other in the Self (IOS) scale, where a higher rating indicated more closeness to the self (Aron et al., 1992). Additionally, we checked whether indicated closeness correlated with behavior in the task for the friend and unknown other. Lastly, we performed subject level checks on discounting behavior by assessing the discounting curve for each individual separately and testing for non-systematic data (Johnson and Bickel, 2008).

To examine specific effects of delay magnitude and immediate rewards we performed separate linear mixed effects models with delay and immediate reward as a fixed effect, normalized SVs as the dependent variable, and subject and family ID as random effects to account for repeated measures and dependency within twins. To assess whether the effects differed by delay-beneficiary we added delay-beneficiary as a fixed effect. Pairwise comparisons were examined with paired t-tests.

For delay magnitude, we first compared SVs for all delays (SV ∼ delay) for each delay-beneficiary, after which we also compared SV over delay-beneficiaries (SV ∼ delay-beneficiary) for each delay. We used Bonferroni correction per delay-beneficiary or delay to correct for multiple comparisons. The same method was applied for immediate rewards, examining pairwise comparisons of SV by immediate rewards (SV ∼ Immediate reward) for each delay-beneficiary, and by delay-beneficiaries (SV ∼ delay-beneficiary) for each immediate reward. We additionally applied Bonferroni correction per delay-beneficiary or immediate reward.

Next, we assessed the effect of delay-beneficiary on AUC with a linear mixed effects model with AUC as the dependent variable and delay-beneficiary as a fixed effect. Random effects of subject and family ID were added to account for repeated measures and dependency within twins. Age was hierarchically added as a fixed effect to examine age effects.

Where available, Bayes factors are provided to provide additional evidence. Unfortunately, Bayes factors are not yet available for mixed linear models (version 0.18.3, JASP Team, 2024). Default priors were used to increase the objectivity and reproducibility of the Bayes factor values. These priors reflect plausible effect sizes in psychology (Keysers et al., 2020). Because for the Bayesian correlation analysis, the default setting in JASP is a non-informative prior, we checked whether conclusions would change if using a weakly or moderately informative prior. We found that conclusions remained consistent in all but one case, in which the BF_10_ switches from moderate (BF_10_=0.298) to anecdotal (BF_10_=0.684, see Table A.5). In that case we mentioned this fact in the relevant result section.

### 2.6 MRI data analysis

#### 2.6.1 Preprocessing

MRI preprocessing was performed in SPM12 (Welcome Department of Cognitive Neurology, London, United Kingdom). Functional scans were preprocessed using multiple steps. First, they were corrected for slice time acquisition and motion using re-alignment (with 6 parameters). Anatomical scans were then normalized to the MNI 305- stereotaxic space using a 12-parameter affine non-linear transformation involving cosine basic functions (Cocosco et al., 1997). Functional scans were then registered to the template using the anatomical scans. Volumes of all participants were resampled to 3-mm cubic voxels.

Lastly, smoothing was applied using a 6 mm FWHM isotropic Gaussian Kernel.

#### 2.6.2 First level analysis

First level individual analyses were performed using a general linear model in SPM12. The fMRI time series were modeled as zero duration events time locked to the onset of the choice screen. We classified events based on the choice of the participant, splitting the 3 different conditions into 6 experimental events modeled as separate predictors (‘Self – Immediate’, ‘Self – Delay’, ‘Self/Friend - Immediate’, ‘Self/Friend - Delay’, ‘Self/Other - Immediate’, ‘Self/Other - Delay’). In addition, control trials were modeled as a single event and predictor independently of choice (‘Control’). All predictors were then convolved with the hemodynamic response function (HRF). The six motion regressors were added as covariates along with a high pass filter of 120 Hz. Trials on which participants did not respond were marked as invalid and were excluded from analysis. We used least squares parameter estimates (Pes) for each predictor of interest. These resulted in individual contrast images that were used in group level analysis.

#### 2.6.3 Second level analysis

To investigate the effects of the experimental task, we first created a contrast of all choices versus the control condition for which results can be found in Appendix C. We then performed a whole brain ANOVA of delay-beneficiary (self, friend, or unknown other) by choice (immediate or delay) using the experimental conditions. We performed pairwise comparisons of brain activation using paired sample t-tests in regions resulting from the ANOVA. Follow up statistical analyses on ROIs extracted from the factorial ANOVA were only performed for post-hoc testing and to visualize the patterns. We therefore do not report the F-values from the interaction effects, as this would be circular.

We included bayes factors derived from paired sample t-tests using JASP (version 0.18.3, JASP Team, 2024). Finally, we examined the effect of participants’ AUC values on brain activity by performing a whole brain multiple regression using the AUC values for every delay-beneficiary. All contrasts were added to Neurovault (https://neurovault.org/collections/TARDMHYT/), and all analyses are reported with exact p-values in Appendix C.

#### 2.6.4 Heritability analyses

To explore to what extent genetics and environmental influences contribute to delay discounting behavior, we first performed within-twin pair Pearson correlations for AUC values, separately for both monozygotic (MZ) and dizygotic (DZ) twins. We calculated Pearson correlations for overall AUC values, and additionally for AUC values by delay-beneficiary. In the case of a genetic influence, correlations for MZ twins will be higher compared to correlations for DZ twins. If correlations are also high for DZ twins, this indicates an influence of shared environment. To assess whether correlations for MZ and DZ twins differed, we compared them using a method for independent samples derived from Lenhard and Lenhard (2014).

We then performed structural equation full ACE models for overall AUC, as well as AUC separated by delay-beneficiary (Neale et al., 2016). This allowed us to get estimates of additive genetic factors (A), shared environmental factors (C), and unique environment or measurement error (E). As all twins included in the study shared a 100 % of their environment, the correlation for shared environment (C) was set to 1 for all twins. The correlation for the genetic factor (A) was set to 1 for monozygotic twins and to 0.5 for dizygotic twins.

The final sample used for the heritability analyses included 96 complete twins (55 monozygotic twins and 41 dizygotic twins). Although we initially included 196 adolescents (98 pairs), for two twin pairs only one individual from each pair had complete data. They were therefore excluded from the heritability analyses, resulting in 96 complete twins. Zygosity was determined through buccal samples that were collected using mouth swabs (Whatman Sterile Omni Swab).

## 3 Results

### 3.1 Behavioral results

#### 3.1.1 Manipulation checks

A total of 193 participants completed the exit questionnaire containing the questions for the manipulation checks. One of these individuals did not complete the question about uncertainty of payout, resulting in a total of 192 participants for that analysis. For the closeness ratings, two individuals did not complete the question for the friend, and three individuals did not complete the question for the unknown other, resulting in a participant sample of 191 and 190 correspondingly.

Manipulation checks showed that overall participants were not worried whether they would receive the payment (*M* = 2.44, *SD* = 1.77, *N* = 192). Uncertainty did not significantly correlate with the AUC values (Self: *r* =.11, *p* =.12, BF_10_ = 0.298; Friend: *r* =.06, *p* =.379, BF_10_ = 0.133; Other: *r* =.07, *p* =.317, BF_10_ = 0.149, *N* = 192).

Because the Bayesian correlation analyses use a non-informative prior (Streched Beta Prior Width SBPW = 1), to check for the robustness, we repeated the analyses with a weekly (SBPW = 0.5) and moderately informative prior (SBPW = 0.2). For the delay-beneficiary self, we obtained BF_10_ = 0.439 and BF_10_ = 0.684 respectively (Table A.5). The BF_10_ switches to 0.684 if we use a more informative prior, suggesting that this level of correlation should not be taken as evidence for the absence of a small association. For the delay-beneficiary friend, we obtained BF_10_ = 0.197 and BF_10_ = 0.315 respectively (Table A.5). For the delay-beneficiary unknown other, we obtained BF_10_ = 0.221 and BF_10_ = 0.351 respectively (Table A.5). Bayes factors for the delay-beneficiary friend and unknown other remain below or similar to 1/3 and can therefore be considered evidence that uncertainty about the payment did not influence behavior in the task for the delay-beneficiary friend and unknown other.

**Fig. 2:**
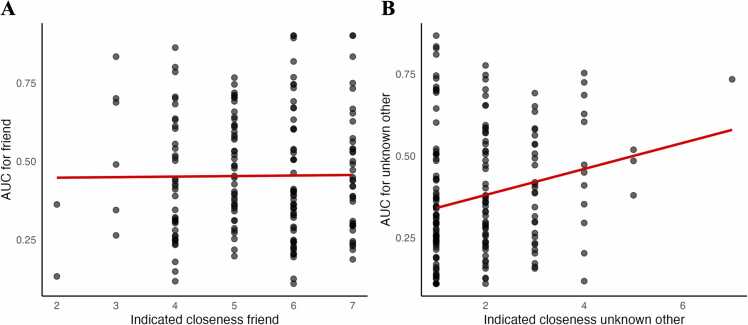
Correlations between AUC values and indicated closeness for the delay-beneficiary friend (*r* = .01, *p* = .885) (A) and unknown other (*r* = .22, *p* = .002) (B).

Regarding IOS closeness ratings, participants reported their friends (*M* = 5.4, *SD* = 1.18, *N* = 191) to be significantly closer compared to unknown others (*M* = 1.88, *SD* = 1.07, *N* = 190), as indicated by a paired sample t t-test, *t*(189) = −31.43, *p* <.001, BF_10_ = 2.017⋅10+73. Indicated IOS closeness of the friend did not correlate with AUC values when friend was the delay-beneficiary (*r* =.011, *p* =.885, BF_10_ = 0.09, *N* = 191, Fig. 2A).

Indicated IOS closeness of the unknown other significantly correlated with AUC values when unknown other was the delay-beneficiary (*r* =.22, *p* =.002, BF_10_ = 9.4, *N* = 190, Fig. 2B), showing that more closeness was associated with higher AUC value for the unknown other.

Because the Bayesian correlation analyses use a non-informative prior (Streched Beta Prior Width SBPW = 1), to check for the robustness, we repeated the analyses with a weekly (SBPW = 0.5) and moderately informative (SBPW = 0.2). Results for the delay-beneficiary friend show BF_10_ = 0.137 and BF_10_ = 0.221 respectively, providing evidence for the absence of an effect of indicated closeness of the friend on behavior for the delay-beneficiary friend (Table A.5). The same robustness check was applied for the delay-beneficiary unknown other, yielding BF_10_ = 13.371 and BF_10_ = 18.817 respectively (Table A.5). These results provide evidence for an effect of indicated closeness of the unknown other on behavior for the delay-beneficiary unknown other.

Due to the appearance of an outlier in Fig. 2B (one individual indicating maximum closeness with the stranger), we tested for influential cases using the cook’s distance. None of the values was above 1, indicating that none of the cases was of excessive influence (*M* = 0.005, *SD* = 0.008, range = 0.00–0.06, *N* = 190, Figure B.1).

Lastly, we performed subject level checks on discounting behavior to assess if delay discounting was observed at the subject level. We assessed the discounting curve for each individual separately and visually inspected all graphs, after which we applied the algorithm by Johnson and Bickel (2008) to check for nonsystematic data. For most individuals, we observed delay discounting at the subject level. Although some individual patterns showed a small violation of criterion 2, we did not remove these participants from our analyses due to the exploratory nature of the current study and the newly introduced delay-beneficiaries, which may explain small deviations from criterion 2. All results can be found in Table B.2 and Figure B.2.

#### 3.1.2 Effects of delay magnitude on delay discounting for different delay-beneficiaries

To assess the effect of delay magnitude on decisions for the three delay-beneficiaries, we performed a linear mixed effects model of delay-beneficiary and delay as fixed effects on normalized SV values as the dependent variable, including random effects for subject and family ID to account for repeated measures and dependency within twins. This resulted in the following model: SV ∼ Condition*Delay + (1|Subject) + (1|FamilyID). We found a main effect of delay *F(*3, 2145) = 487.34, *p* <.001, η^2^_p_ = 0.41) as well as a main effect of delay-beneficiary *F(*2, 2145) = 312.38, *p* <.001, η^2^_p_ = 0.23, and an interaction between delay and delay-beneficiary, *F(*6, 2145) = 10.94, *p* <.001, η^2^_p_ = 0.03. The interaction showed that the effect of delay differed by delay-beneficiary, such that the decrease of SV with increasing delays was strongest for the self, then for the friend, and weakest for the unknown other (Figure B.3 A).

**Fig. 3:**
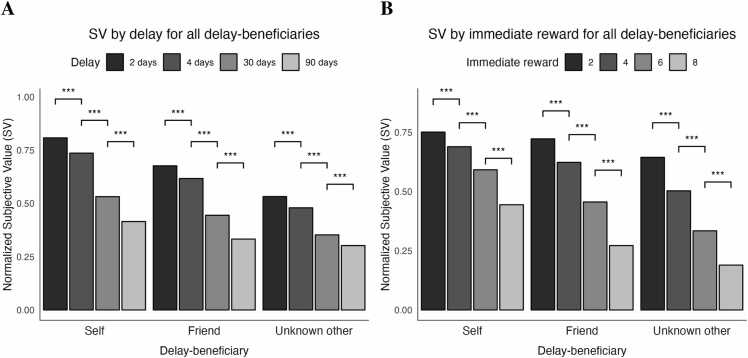
Normalized SVs for all three delay-beneficiaries by delay magnitude (A) and immediate reward (B). Note. *** denotes a p-value <.001.

Pairwise comparisons showed that the normalized SV decreased monotonically with increasing delay magnitude for each delay-beneficiary (all *p’s* <.001, Fig. 3A). Additionally, pairwise comparisons showed that for almost all delay magnitudes, normalized SV decreased significantly over delay-beneficiaries (*p’s* <.001). Only for the delay of 90 days for the friend compared to the unknown other, no significant difference was found (*p* =.059), but the Bayes factor confirms that this is not evidence for the absence of an effect (BF_10_ = 1.178).

#### 3.1.3 Effects of immediate reward on delay discounting for different delay-beneficiaries

To assess the effect of immediate reward on decisions for the three delay-beneficiaries, we performed a linear mixed effects model of delay-beneficiary and immediate reward as fixed effects on normalized SV values as the dependent variable, including random effects for subject and family ID to account for repeated measures and dependency within twins. This resulted in the following model: SV ∼ Condition*Immediate reward + (1|Subject) + (1|FamilyID). We found a main effect for immediate reward *F*(1, 2151) = 1908.77, *p* <.001, η^2^_p_ = 0.47, as well as a main effect for delay-beneficiary *F(*2, 2151) = 279.96, *p* <.001, η^2^_p_ = 0.21, as well as an interaction between immediate reward and delay-beneficiary, *F(*2, 2151) = 29.73, *p* <.001, η^2^_p_ = 0.03. The interaction showed that the effect of immediate rewarddiffered by delay-beneficiary, such that the decrease of SV with increasing immediate reward was stronger for friend and unknown other compared to the self (Figure B.3B).

Pairwise comparisons showed that the normalized SV decreased monotonically with increasing immediate reward magnitude for each delay-beneficiary (all *p’s* <.001, Fig. 3B). Additionally, pairwise comparisons showed that for almost all immediate rewards, the normalized SV decreased over delay-beneficiaries (all *p’*s <.001). Only for the immediate reward of 2 for the self compared to the friend as delay-beneficiary the p-value was >.001, although still significant after correcting for multiple comparisons (*p* =.017).

#### 3.1.4 Effects of delay-beneficiary on delay discounting

To assess effects of delay-beneficiary (self, friend, unknown other) on delay discounting we performed a linear mixed effects model of delay-beneficiary as a fixed effect on AUC values, including random effects for subject and family ID to correct for repeated measures and dependency within twins. This resulted in the following model: AUC ∼ Condition + (1|Subject) + (1|FamilyID). Participants had an average AUC score of .46 (*SD* = 0.22, *N* = 196). AUC scores differed significantly between the three delay-beneficiaries (*F*(2, 390) = 104.69, *p* <.001, η^2^_p_= 0.35).

**Fig. 4:**
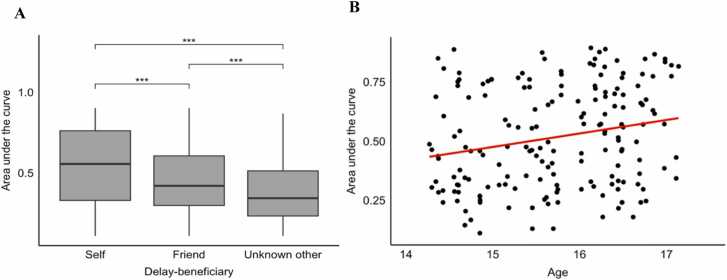
*AUC and Age-effect. Note*. (A) Area under the curve (AUC) scores by task condition (self now – self later; Self, self now – friend later; Friend, self now – unknown other later; Unknown other). *** denotes a p-value <.001. (B) Area under the curve (AUC) in the self condition plotted with age. The graph shows that older adolescents show higher AUC scores with increasing age, suggesting older adolescents make more delayed choices.

Pairwise comparisons showed that the AUC was highest when the delay-beneficiary was self (*M* = 0.55, *SD* = 0.23), then for the friend (*M* = 0.46, *SD* = 0.19), and lowest for the unknown other (*M* = 0.38, *SD* = 0.19), indicated by a paired sample t tests of friend compared to self *t*(195) = −8.74, *p* <.001, friend compared to unknown other *t*(195) = 7.62, *p* <.001, and self compared to unknown other *t*(195) = 12.06, *p* <.001 (Fig. 4A). This indicates that when all other factors are kept constant, i.e., the immediate option always benefits the self, and the delays for the delayed beneficiary are the same, participants choose the delay more when it benefits self than friend than an unknown other.

To assess the effect of age on AUC score, we added age as a fixed effect, resulting in the following model: AUC ∼ Condition + Age + (1|Subject) + (1|FamilyID). Adding a a linear effect of age showed a significant effect for age *F*(1, 97.94) = 4.04, *p* =.047, η^2^_p_=.04.

We then separated effects by delay-beneficiary by performing separate linear mixed effect models for each condition, removing the random effect for subject. This resulted in the following model for all three conditions: AUC ∼ Age + (1|FamilyID). Only a significant effect for the self remained (*F*(1, 97.95) = 4.44, *p* =.038, η^2^_p_=.04), see Fig. 4B. The same analyses were not significant for friend (*F*(1, 97.92) = 2.41, *p* =.124, η^2^_p_=.02) and unknown other (*F*(1, 97.83) = 2.79, *p* =.098, η^2^_p_=.03).

### 3.2 fMRI results

#### 3.2.1 Whole brain ANOVA of target by choice

A whole brain ANOVA of target by choice was performed for participants who had observations in each of the six cells (delayed and immediate choices for self, friend, and unknown other). In total, 18 individuals showed empty cells and were excluded from this analysis. An overview of all subjects’ choices by condition can be found in Figure C.1.

The sample with complete MRI data therefore resulted in 138 adolescents (50,7 % females, *M*_age_ = 15.80, *SD*_age_= 0.78, age range = 14.27 – 17.14).

The main effect of choice (FWE and FDR cluster corrected, *k* = 123, with initial uncorrected *p* =.001) resulted in activation in the visual cortex and the left amygdala (Figure C.3). Given our main interest in social, emotional, and cognitive processing related to delay discounting, we extracted activation of the observed left amygdala cluster masked with the anatomical amygdala from the AAL atlas and performed follow up testing using a paired sample t-test to examine the specific effect for the different choices. This showed increased activity in the left amygdala for delayed relative to immediate choices *t*(137) = 4.46, *p* <.001, BF_10_ = 859.77 (Figure C.3). The whole brain contrast can be found on neurovault (https://neurovault.org/collections/TARDMHYT/images/807530/).

**Fig. 5:**
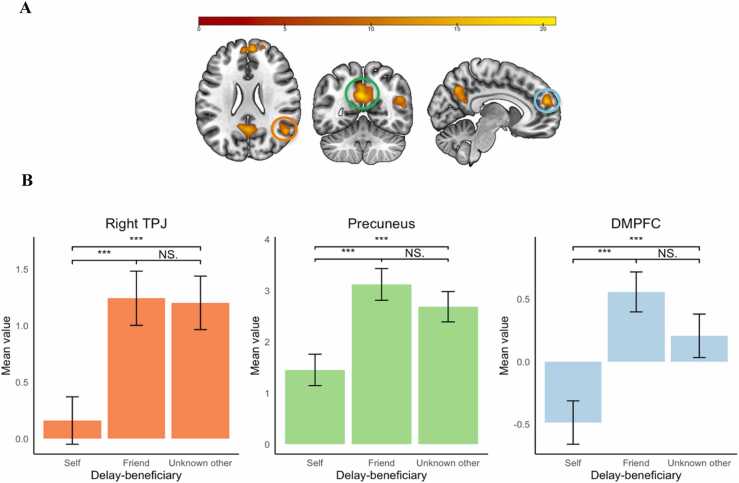
The main effect of delay-beneficiary in the right temporal-parietal junction (TPJ), precuneus, and dorsal medial prefrontal cortex (DMPFC). Note. FWE cluster corrected (*k* = 238) with initial uncorrected p-value (*p* =.001). The brain regions in A are indicated with corresponding colors to the graphs displayed in B.

The main effect of target (FWE cluster corrected, *k* = 238, with initial uncorrected *p* =.001) resulted in three regions that were differentially activated: the right temporal-parietal junction (TPJ), precuneus, and dorsal medial prefrontal cortex (DMPFC; Fig. 5). We extracted activation for these clusters and performed follow up testing using paired sample t-tests to examine the specific effects for the delay beneficiaries. These analyses showed that for all three regions, activation was significantly higher for the friend than for the self (right TPJ: *t*(137) = −5.09, *p* <.001, BF_10_ = 11018.76, precuneus: *t*(137) = −5.53, *p* <.001, BF_10_ = 72626.98, DMPFC: *t*(137) = −6, *p* <.001, BF_10_ = 611269.71). The same pattern was observed when comparing the self to the unknown other, showing higher activity for the unknown other compared to the self (Right TPJ: *t*(137) = −4.44, *p* <.001, BF_10_ = 787.26, precuneus: *t*(137) = −3.6, *p* <.001, BF_10_ = 41.24, DMPFC: *t*(137) = −3.4, *p* <.001, BF_10_ = 22.08). Additionally, for all three regions, no significant difference was found for the unknown other compared to the friend (DMPFC: *t*(137) = 1.86, *p* =.06, BF_10_ = 0.51, precuneus: *t*(137) = 1.36, *p* =.17, BF_10_ = 0.23, right TPJ: *t*(137) = 0.18, *p* =.86, BF_10_ = 0.1). The bayes factor however suggest rather weak evidence for no effect in the DMPFC. The whole brain contrast for the main effect of target can be found on neurovault (https://neurovault.org/collections/TARDMHYT/images/807529/).

**Fig. 6:**
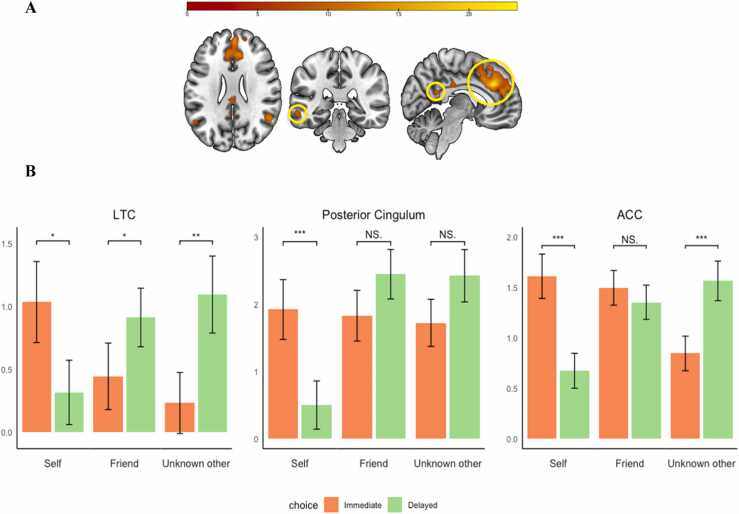
Interaction of delay-beneficiary (self, friend, unknown other) with choice (immediate or delay) for left temporal cortex (LTC), posterior cingulum, and anterior cingulate cortex (ACC). Note. FWE cluster corrected (k = 139) with initial uncorrected p-value (p =.001). Regions are indicated in consequent order of graph presentation.

The interaction between target and choice (FWE cluster corrected, *k* = 139, with initial uncorrected *p* =.001) resulted in activation in a network of regions including the right dorsolateral prefrontal cortex (DLPFC), bilateral insula, pre-motor medial prefrontal cortex/anterior cingulate cortex (ACC), bilateral temporal-parietal junction (TPJ), posterior cingulum, posterior cingulate cortex (PCC), and the left temporal cortex (LTC; Table 2 and Fig. 6A). We extracted activation of all clusters and performed follow up testing using paired sample t-tests to examine the specific effects for the delay-beneficiaries and choices.

**Table 1:** *MNI coordinates of local maxima activated for the main effect of delay-beneficiary*.

Area of activation	MNI coordinates			Test statistic	Cluster Size
*Delay-beneficiary*	x	y	z	*F*	
Precuneus	−4	−58	30	21.25	931
Dorsal medial prefrontal cortex (DMPFC)	8	56	20	17.49	4111
Right temporal-parietal junction (TPJ)	50	−58	24	14.92	258

**Table 2:** MNI coordinates of local maxima activated for the interaction between delay-beneficiary and choice.

Area of activation	MNI coordinates			Test statistic	Cluster Size
*Delay-beneficiary by Choice*	x	y	z	*F*	
Anterior Cingulate Cortex/ Premotor Cortex (ACC)	6	32	32	23.89	3653
Right temporal-parietal junction (TPJ)	52	−54	44	14.73	437
Posterior Cingulum	−2	−48	20	12.92	143
Right Inferior Frontal Cortex / Insula	44	22	−12	12.81	406
Left inferior Frontal Cortex / Insula	−40	22	−8	12.55	327
Left temporal parietal junction (TPJ)	−44	−58	36	12.11	384
Right dorsal lateral prefrontal cortex (DLPFC)	36	20	38	11.64	150
Left Temporal Cortex (LTC)	−62	−34	−10	10.85	140
Posterior Cingulate Cortex (PCC)	−2	−24	30	10.20	139

These analyses resulted in three data patterns. We visualized one region for each pattern in Fig. 6B, all other results can be found in Table C.3. First, only the LTC showed significant differences between now and later for all delay-beneficiaries. In this region, activation was significantly higher for immediate choices for self when the delay-beneficiary was self (*p* <.05), whereas when the delay-beneficiary was friend or unknown other, the activation pattern was reversed, with higher activation for delay than for immediate choices (*p* <.05 and <.01 respectively). Second, in the PCC and posterior cingulum, activation was significantly higher for immediate choices for self when the delay-beneficiary was self (both *p*’s <.001), but the choice differences were not significant when the delay-beneficiary was friend or unknown other. Third, in the bilateral insula, the ACC, the right DLPFC, and the bilateral TPJ, activation was significantly higher for immediate choices for self when self was the delay-beneficiary (all *p*’s <.05). Activation was not significantly different for immediate versus delayed choices when friend was the delay-beneficiary (all *p*’s n.s.), whereas when the delay-beneficiary was the unknown other, the activation pattern was reversed, with higher activation for delayed than for immediate choices (all *p*’s <.01). The whole brain contrast of the interaction effect can be found on neurovault (https://neurovault.org/collections/TARDMHYT/images/807531/).

#### 3.2.2 Whole brain regression with AUC

**Fig. 7:**
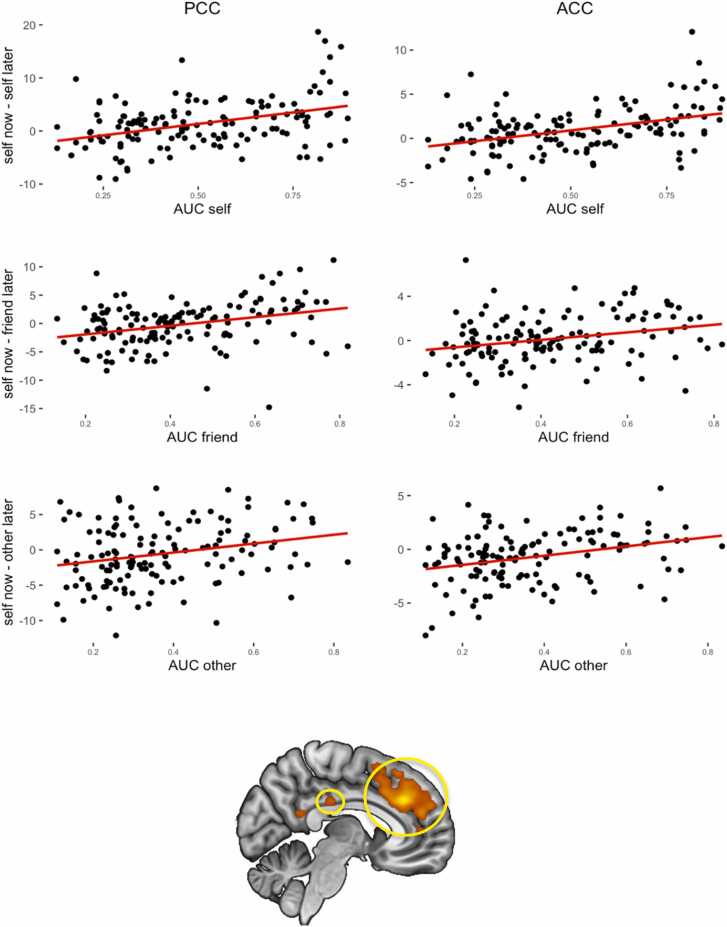
*Results for the regression with AUC values in the posterior cingulate cortex (PCC) and anterior cingulate / premotor cortex (ACC). Note.* Correlations between brain activity for now – later for the regions in the regression analysis that showed overlap with the interaction effect. Areas are indicated in consequent order and are derived from the interaction effect. AUC shows a positive association with brain activity, such that when activity for a decision now is higher than activity for a decision later within these regions, individuals have higher AUC overall values.

To examine whether activation in the various contrasts correlated with AUC, we performed whole brain regression analyses for each contrast (self now – self later; self now – friend later; self now – unknown other later) with AUC as a regressor. The analyses were performed for all participants with data for the specific contrasts (thus excluding participants with empty cells per contrast). The first analysis was performed on the self now – self later contrast with self later AUC as regressor (FWE cluster corrected, *k* = 180, with initial uncorrected *p* =.001, *N* = 141). The second analysis was performed on the self now – friend later contrast with friend later AUC as regressor (FWE cluster corrected, *k* = 299, with initial uncorrected *p* =.001, *N* = 154), and we performed the third analysis on the self now – unknown other later contrast with unknown other later AUC as regressor (FWE and FDR cluster corrected, *k* = 184, with initial uncorrected *p* =.001, *N* = 148). All analyses showed activation in a similar network of regions that was positively correlated with AUC values, such that higher activity for now versus later in these regions is positively related to AUC value. The whole brain contrasts are uploaded to neurovault (self; http://neurovault.org/collections/TARDMHYT/images/807526/,

friend; http://neurovault.org/collections/TARDMHYT/images/807527/, unknown other; http://neurovault.org/collections/TARDMHYT/images/807528/). Fig. 7 illustrates the relationship between brain activity for now – later with AUC in two regions that showed overlap with the network identified in the interaction effect as a result of visual inspection (Posterior Cingulate Cortex, Anterior Cingulate cortex/ premotor cortex; Fig. 7).

### 3.3 Heritability estimates

To test for the contributions of genetic (A), shared environment (C) and unique environment/measurement error (E) on AUC, we first calculated within twin Pearson correlations for AUC values, both overall and for all delay-beneficiaries separately (Table 3 and Figure C.5). For MZ twins, we found significant correlations for all AUC measures except when the delay-beneficiary was unknown other (*p* =.14). For DZ twins, we found significant correlations for all AUC measures. Correlations were high for both MZ and DZ twins and did not differ significantly in any of the AUC measures except for the AUC when the delay-beneficiary was self, with a higher correlation for DZ twins (*Z* = −1.95). This indicates a low influence of genetics, as twins with higher genetic similarity show lower or comparable similarity in behavior. Additionally, as both groups show high correlations in behavior, this indicates an influence of shared environment.

**Table 3:** Pearson correlations between twin pairs for monozygotic and dizygotic twins and the contributions of genetics (A factor), shared environment (C factor), and unique environment/measurement (E factor) to delay discounting behavior.

Outcome		MZ	DZ	Z	Model	A	C	E
AUC overall	*r*	.31	.47	−0.89	ACE	0.00	0.37	0.63
	*p*	.02	.002		CI 95 %	[0.00, 0.50]	[0.00, 0.53]	[0.45, 0.81]
AUC self	*r*	.27	.60	−1.95*	ACE	0.00	0.39	0.61
	*p*	.05	<.001		CI 95 %	[0.00, 0.27]	[0.12, 0.55]	[0.45, 0.79]
AUC friend	*r*	.29	.45	−0.87	ACE	0.00	0.35	0.65
	*p*	.03	.003		CI 95 %	[0.00, 0.51]	[0.00, 0.51]	[0.45, 0.84]
AUC unknown other	*r*	.20	.34	−0.71	ACE	0.00	0.27	0.73
	*p*	.14	.03		CI 95 %	[0.00, 0.46]	[0.00, 0.44]	[0.52, 0.93]

**Fig. 8:**
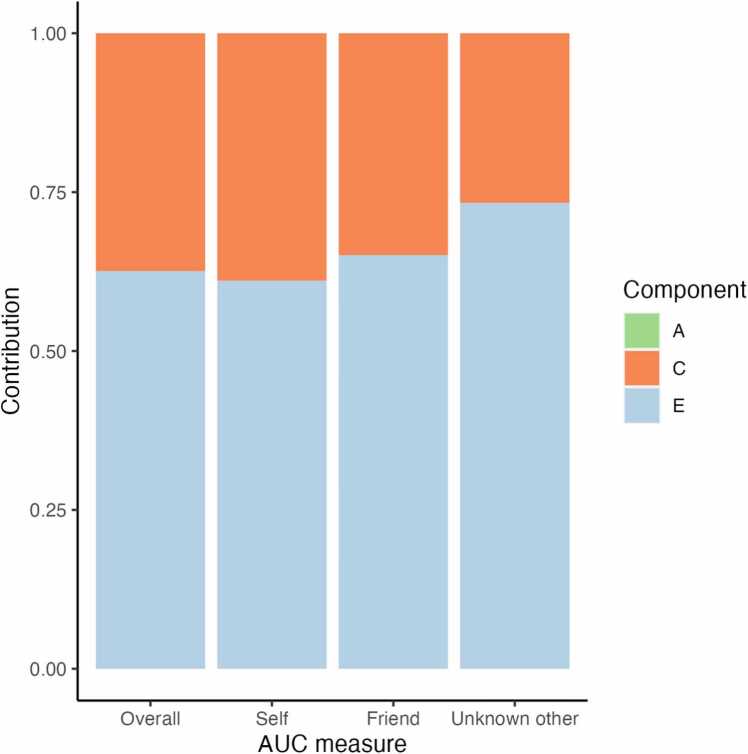
*Contributions of genetics (A factor), shared environment (C factor), and unique environment/measurement error (E factor) to delay discounting behavior in the ACE model*.

We then fitted the full ACE models (*N* = 96; 55 MZ twins and 41 DZ twins; Table 3). The analyses showed that variation in overall AUC was explained by a combination of shared environment and unique environment/measurement error (C = 37 %, E = 63 %). For AUC with self as the delay-beneficiary, variation was also explained by a combination of shared environment and unique environment/measurement error (C = 39 %, E = 61 %). For AUC with friend as the delay-beneficiary, variation was additionally explained by a combination of shared environment and unique environment/measurement error (C = 35 %, E = 65 %). Lastly, when the delay-beneficiary was the unknown other, variation was explained by a combination of shared environment and unique environment/measurement error (C = 27 %, E = 73 %). These results indicate a moderate effect of shared environment, while there seems to be no effect of genetics on all AUC measures (Fig. 8). We additionally performed Bayesian ANOVAs of zygosity for all AUC measures to assess whether genetic effects are likely absent. These analyses showed substantial evidence that AUC values do not differ between monozygotic and dizygotic twins for the task overall and when the delay-beneficiary is a friend or unknown other (BF_10_ = 0.213, BF_10_ = 0.174, BF_10_ = 0.157, correspondingly). When the delay beneficiary is self, the bayes factor indicates anecdotal evidence for no effect (BF_10_= 0.343).

## 4 Discussion

The aim of this explorative study was to examine the behavioral and neural correlates of balancing immediate rewards for the self against delayed rewards for self or others, specifically friends and unknown others. We observed that participants choose immediate rewards for the self most often if the alternatives were delayed rewards for unknown others, followed by delayed rewards for a friend, and least often if the alternative were delayed rewards for self, as reflected by significant pairwise differences in the Area-Under-the-Curve. Analyses for the subjective values showed that the effect of increasing delays was strongest if delayed rewards were for self, and weakest when delayed rewards were for unknown others, with friends presenting an intermediate slope. At the neural level, we observed that regions typically associated with delay discounting choices (van de Groep et al., 2023), including the medial prefrontal cortex, DLPFC, insula and TPJ, were differentially activated depending on the target, with a shift in more activity for immediate choices for self to more activity for delayed choices for unknown others. Finally, behavioral differences in delay discounting for self, friends and unknown others were correlated with neural activity for immediate versus delayed choices.

The findings of this study align with a substantial body of literature on delay discounting behavior. Consistent with prior studies, adolescents were able to delay gratification to obtain a larger reward in the future. Additionally, the subjective value of the delayed reward decreased with increasing delay magnitude or immediate reward (McClure et al., 2004). We also confirmed an age-related increase in delay of gratification between ages 14–17-years (Peper et al., 2018, Steinberg et al., 2008, van de Groep et al., 2023), showing that adolescents become better at resisting immediate rewards for future benefit. Several explanations may account for this developmental difference, such as the development of cognitive control (Steinberg et al., 2008) and the development of future orientation (van den Bos et al., 2015). A novel element of the current design is that adolescents did not only make future oriented choices for themselves but also for friends and unknown others, where delayed outcomes were always juxtaposed against immediate rewards for self.

The results showed that delay discounting behavior differed depending on the delay-beneficiary. When examining the effect of delay and delay-beneficiary on subjective values, we observed an interaction effect indicating that the effect of delay depends on the delay-beneficiary. The effect was largest for the self, such that when the delay-beneficiary was self, the subjective value decreased most over increasing delay. This effect was somewhat smaller for the friend, and smallest for the unknown other. These results indicate that most delay-discounting occurred when the delay-beneficiary was self, then for the delay-beneficiary friend, and least when the delay beneficiary was the unknown other (Figure B.3).

Whereas the analysis with delay considers the relative difference of SV over delay, the AUC values represent a sum of subjective values and therefore an overall measure of how often an individual chooses the delayed reward. These results show that individuals generally choose the delayed reward more often when the delay-beneficiary is self. The lower AUC observed for friend and unknown other is indicative of generally less delayed reward choices for these delay-beneficiaries. This suggests that individuals generally tend to give less to others than to themselves, consistent with other studies (Guroglu et al., 2014). Future studies could include a measure of baseline giving to control for this effect.

The differential behavioral patterns were mirrored in the patterns of neural activity. First, the general contrast of all choices relative to the control condition (circle matching) revealed a large set of brain regions that are associated with cognitive control and motor planning, confirming the validity of the task (van de Groep et al., 2023). Second, we observed that the amygdala was more activated for delay versus immediate choices across all targets. Prior studies have shown that the amygdala has been associated with decisions under risk or ambiguity (Levy et al., 2010, Hsu et al., 2005). In these studies, participants had to choose a gamble with a known (risk) or unknown (ambiguity) probability, to win a larger reward compared to a smaller certain reward. As delayed rewards are considered more risky and ambiguous (Jiang and Dai, 2021), the higher amygdala activation in delayed choices could reflect this increased risk and ambiguity. Third, when examining the effects of target, we confirmed that especially the midline areas dorsal medial prefrontal cortex and precuneus as well as the right TPJ were more active when thinking about choices that affected others compared to self. We found no difference in neural activity depending on whether the other was a friend or unknown other. These findings suggest that these areas have a general function in thinking about others or self-other differentiation, independent of the closeness of the relationship. The dorsal medial prefrontal cortex has been described in meta-analyses on research in adults as an important region for cognitive perspective taking and thinking about self in relation to others (Denny et al., 2012). Similarly, the precuneus has been associated with mental imagery of others and their perspectives (Schurz et al., 2014). While the functionality for the TPJ is more diverse and dependent on the specific subregion, its overarching function was suggested to lie in inferring mental states of others (Schurz et al., 2014). This study confirms similar roles of these areas in a sample of 14–17-year-old adolescents.

The most important neural differences were observed when comparing immediate versus delayed choices for the three targets: self, friends, and unknown others. In a whole brain analysis, we identified various brain regions that have previously been associated with choice differences (Rilling and Sanfey, 2011), including the pre-motor medial prefrontal cortex/anterior cingulate cortex (ACC), the bilateral insula, the right DLPFC, and the bilateral TPJ. We observed that in these areas, activation was higher for immediate compared to delayed choices when the delay-beneficiary was self. The opposite effect was found when the delay beneficiary was the unknown other, while no effect was observed for the delay-beneficiary friend. These findings suggest a qualitative shift in recruitment of these regions depending on who is the beneficiary.

Possibly, when choosing an immediate reward for self in the context of a delayed reward for self, adolescents need to override the normative choice to delay gratification for self. In contrast, when choosing the delayed reward for an unknown other, adolescents possibly need to override the normative choice to benefit themselves, as choices that benefit unknown others in the future are more uncertain. Such choices may require more cognitive control (often associated with DLPFC activation) and the integration of complex contextual information (often associated with TPJ activation). This could represent a more effortful choice, which we are less willing to make when the decision benefits an unknown other (Lockwood et al., 2017). The finding that the friend as delay-beneficiary shows an intermediate pattern confirms that overriding personal norms are correlated with the relative closeness of the target, consistent with other studies that find differences in behavior depending on the target (Karan et al., 2022, Guroglu et al., 2014, van de Groep et al., 2020). Furthermore, we examined whether individual differences in neural activity were associated with individual differences in behavior. Indeed, we observed that a network of regions was correlated with AUC for all delay-beneficiaries. These findings show that adolescents who showed more activation for immediate choices relative to the delayed choices, chose the delayed reward more often, independent of the delay-beneficiary.

A final question was whether behavior in the delay discounting paradigm could be explained by factors of genetics, shared environment, or unique environment/measurement error. We found that for all measures of AUC, variance was explained by a combination of shared environment and unique environment or measurement error. These findings are in contrast with previous studies that found consistent genetic effects on delay discounting behavior (Anokhin et al., 2011, Anokhin et al., 2015). A potential explanation for the difference in findings could be our relatively small sample size not providing sufficient power to perform an ACE model. Additionally, as we included a slightly different age range, future studies could examine whether contributions of genetic, shared environment and unique environment or measurement error differ across age. However, since this analysis was of exploratory nature, we should be careful in interpreting these effects.

This study also has several limitations that should be addressed in future research. First, the study was not pre-registered and therefore should be interpreted as an exploratory study. Second, the age range was relatively narrow which limited the possibility to test for the transition from childhood to adolescence and from adolescence to adulthood. Therefore, future research should examine the developmental patterns of ingroup-outgroup differentiation in a larger age range. However, the limited age range can also be considered a strength as including a large sample of adolescents from a narrow age range in mid-adolescence can increase robustness of the findings. Third, our sample consists of twins, and although no genetic effects were found and shared environmental effects should be interpreted with caution, including twins introduces dependency within the data that we did not control for in our neural analyses. This could cause our results to be less generalizable to adolescents who grow up without siblings or with siblings of a different age. Fourth, our manipulation of the unknown other could have been interpreted as hypothetical by participants. Although previous research has shown that delay discounting shows similar patterns for hypothetical and real payouts (Bickel et al., 2009, Madden et al., 2004, Scheres et al., 2010), it has not been determined whether this is the case for unknown others. Future studies using a similar design should include a more realistic unknown other such as an unknown participant from the same study, providing information about the age and other demographics of the unknown other. Lastly, while we decided that AUC was an appropriate measure of delay discounting in this study with social beneficiaries as it provides a theory-free measure of delay discounting that does not assume a specific function shape, there are also other measures available which have their own benefits, such as k rates. For example, some studies have found AUC and k rates to have disparate outcomes, which was attributed to a skewed AUC distribution in cases of groups with low or high discounting (Yoon et al., 2017, Mitchell et al., 2015). Future studies could compare approaches to further elucidate which approach is most appropriate for specific beneficiaries. To summarize, this study provides a novel contribution by showing that delay discounting behavior differs for different delay-beneficiaries. These findings provide insight into the mechanisms for youth to make long term decisions that do not only benefit themselves, which is an important aspect of addressing societal issues such as inequality. We demonstrated that neural regions that are important for future orientation are differentially activated depending on who is the beneficiary of the delayed reward. Finally, interactions between the immediacy of choices and beneficiaries imply that adolescents integrate time-related and social-contextual information in their decision-making in a way that may be consistent with their personal norms. These findings provide insights into the neural structures recruited while adolescents balance between the needs of self now and in the future, and the needs of self relative to others.

## Funding

The L-CID study is part of the Consortium on Individual Development (CID; Gravitation grant 2013–2023 awarded by the Dutch Ministry of Education, Culture, and Science, and the Netherlands Organization for Scientific Research, NWO grant number 024.001.003). The GUTS program is funded by an NWO Gravitation programme supported by the Dutch Ministry of Education, Culture and Science of the government of the Netherlands, grant number 024.005.011.

## CRediT authorship contribution statement

**Barbara Braams:** Writing – review & editing, Conceptualization. **Lara Wierenga:** Data curation, Conceptualization. **Michelle Achterberg:** Writing – review & editing, Data curation, Conceptualization. **Suzanne van de Groep:** Writing – review & editing, Writing – original draft, Supervision, Conceptualization. **Eveline Crone:** Writing – review & editing, Writing – original draft, Supervision, Formal analysis, Data curation, Conceptualization. **Lotte H. van Rijn:** Writing – review & editing, Writing – original draft, Visualization, Methodology, Formal analysis, Data curation, Conceptualization. **Lydia Krabbendam:** Writing – review & editing, Supervision, Conceptualization. **Anna van Duijvenvoorde:** Writing – review & editing, Supervision, Conceptualization. **Lucres Nauta-Jansen:** Writing – review & editing, Conceptualization. **Christian Keysers:** Writing – review & editing, Conceptualization. **Berna Guroğlu:** Conceptualization. **Valeria Gazzola:** Writing – review & editing, Conceptualization.

## Declaration of Competing Interest

The authors declare that they have no known competing financial interests or personal relationships that could have appeared to influence the work reported in this paper

## Figures and Tables

**Figure:**
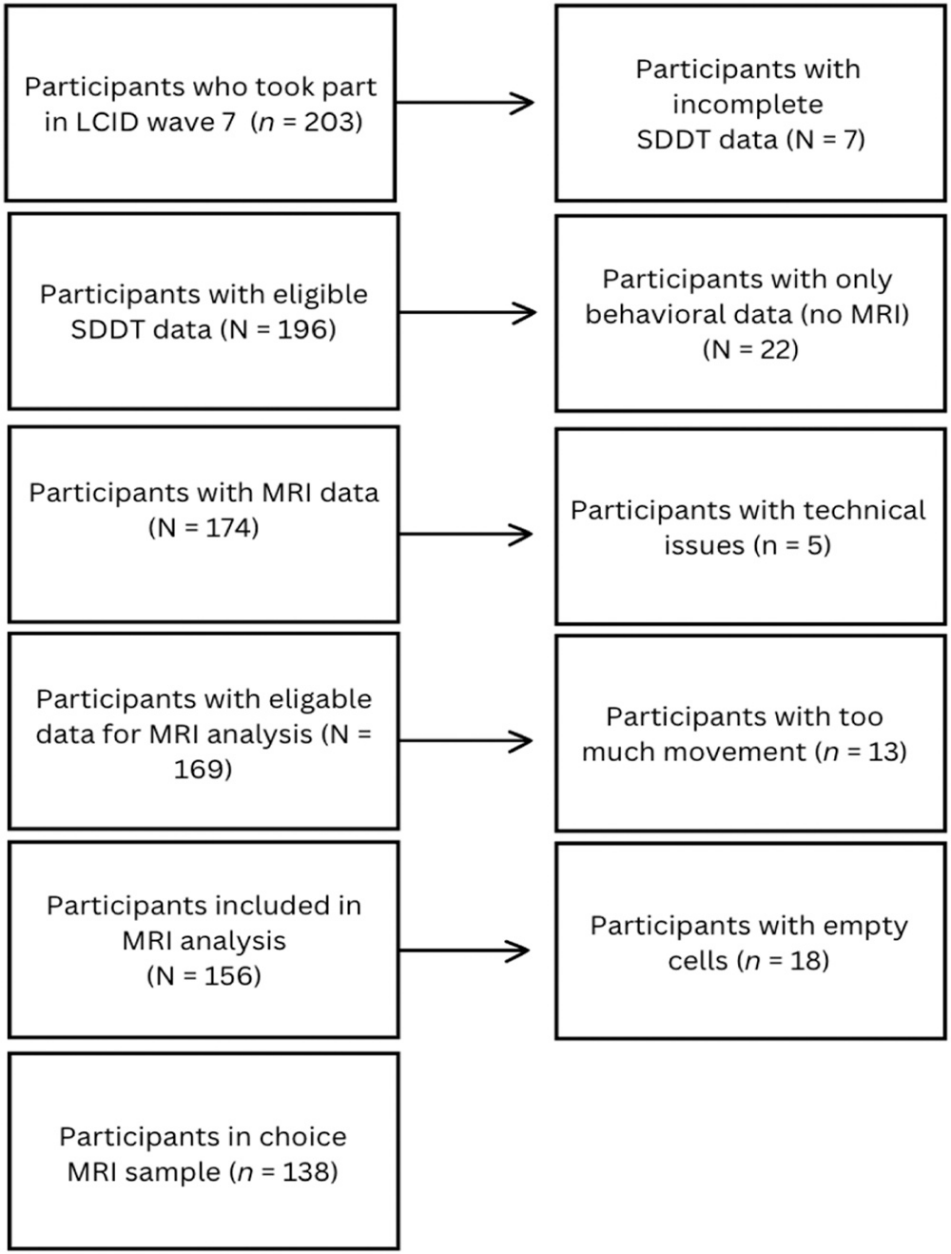
**Figure A.1** Flow chart of participant inclusion in the seventh wave of data collection.

**Table A.1:** Overview of all trial combinations in the Social Delay Discounting Task

Self now- self later	Immediate reward	Delay	Trials	Nr of trials by immediate reward
	2	2	2	
	2	4	2	
	2	30	2	
	2	90	2	8
	4	2	3	
	4	4	3	
	4	30	3	
	4	90	3	12
	6	2	3	
	6	4	3	
	6	30	3	
	6	90	3	12
	8	2	2	
	8	4	2	
	8	30	2	
	8	90	2	8
Self now - friend later				
	2	0	2	
	2	2	2	
	2	4	2	
	2	30	2	
	2	90	2	10
	4	0	2	
	4	2	2	
	4	4	2	
	4	30	2	
	4	90	2	10
	6	0	2	
	6	2	2	
	6	4	2	
	6	30	2	
	6	90	2	10
	8	0	2	
	8	2	2	
	8	4	2	
	8	30	2	
	8	90	2	10
Self now- unknown other later				
	2	0	2	
	2	2	2	
	2	4	2	
	2	30	2	
	2	90	2	10
	4	0	2	
	4	2	2	
	4	4	2	
	4	30	2	
	4	90	2	10
	6	0	2	
	6	2	2	
	6	4	2	
	6	30	2	
	6	90	2	10
	8	0	2	
	8	2	2	
	8	4	2	
	8	30	2	
	8	90	2	10
Control	**Left option**	**Right option**	**Trials**	**Nr of trials**
	O	o	6	
	o	O	6	12

**Table A.2:** Movement before excluding participants (N = 169)

*Measures*	*Mean*	*Run 1*	*Run 2*
Mean (SD)	.11 (.14)	.10 (.12)	.12 (.18)
Minimum	.003	.001	.001
Maximum	7.82	5.53	11.60

**Table A.3:** Movement after excluding participants with >.3 mm maximum movement in any direction (x, y, z, N = 156)

*Measures*	*Mean*	*Run 1*	*Run 2*
Mean (SD)	.10 (.07)	.093 (.07)	.107 (.09)
Minimum	.003	.001	.001
Maximum	2.62	2.71	2.86

**Table A.4:** Movement in the final sample for MRI analysis (N = 138)

*Measures*	*Mean*	*Run 1*	*Run 2*
Mean (SD)	.10 (.05)	.091 (.05)	.106 (.06)
Minimum	.003	.001	.002
Maximum	2.62	2.71	2.86

**Table A.5:** Sensitivity analysis for Bayesian correlation analyses

*Variable 1*	*Variable 2*	*Correlation*	^*BF*^_^*10*^_^*with SBPW*^ =^*1*^	^*BF*^_^*10*^_^*with SBPW*^ =^*0.5*^	^*BF*^_^*10*^_^*with SBPW = 0.2*^
Uncertainty	AUC for self	0.112	0.298	0.439	0.684
Uncertainty	AUC for friend	0.064	0.133	0.197	0.315
Uncertainty	AUC for unknown other	0.073	0.149	0.221	0.351
Closeness friend	AUC for friend	0.011	0.092	0.137	0.221
Closeness unknown other	AUC for unknown other	0.221	9.4	13.371	18.817

**Figure:**
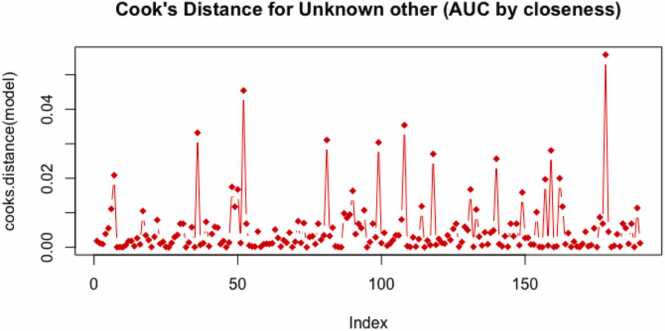
**Figure B.1** Cook’s distance for the relationship between AUC and indicated closeness for the unknown other.

**Table B.1:** Results of the paired sample t-test between closeness ratings for the friend and unknown other

Measure 1	Measure 2	t	df	p	BF10
Relation other	Relation friend	−31.43	189	5.653×10^−77^	2.017×10^+73^

**Table B.2:** Subjects identified according to the second criterion to assess nonsystematic data (Johnson and Bickel, 2008)

Subject	Criterion 2
7	−0027
30	0
63	−0033
82	0
94	−0033
97	0
98	0
118	−0053
131	0
132	−0033
153	0
168	−0007
171	−0033
172	0
183	0

*Note.* Due to differences in delay magnitude, we applied an adjusted criterion of normative subjective value for a delay of 2 days – the subjective value of a delay of 90 days to be smaller or equal to zero.

**Figure:**
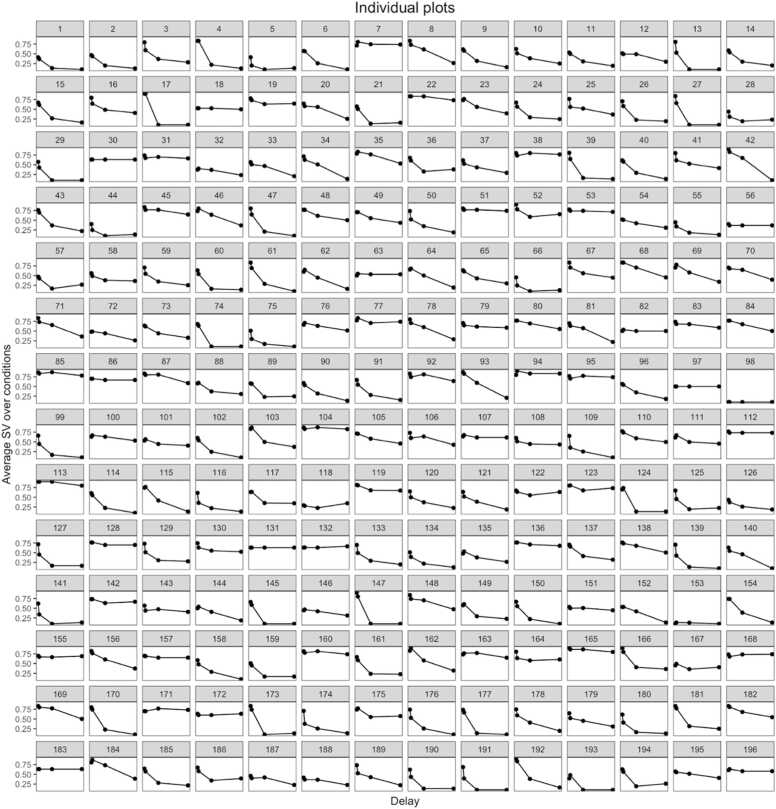
**Figure B.2** Visualization of individual behavioral patterns showing normative subjective value by delay.

**Table B.3:** Results of the linear mixed effects model of normative subjective values by delay and delay-beneficiary

	DF	DF.res	F	p	p<.05	ges
Delay-beneficiary	2	2145	312.38	< 2.2e−16	***	0.23
Delay	3	2145	487.34	< 2.2e−16	***	0.41
Delay-beneficiary:delay	6	2145	10.94	4.902e−12	***	0.03

**Table B.4:** Pairwise comparisons per delay-beneficiary for all delay magnitudes

y	group1	group2	n1	n2	statistic	df	p	p.adj	signif	condition
SV	2 days	4 days	196	196	7.84	195	2.85e−13	1.71e−12	***	Self
SV	2 days	30 days	196	196	15.34	195	2.37e−35	1.42e−34	***	Self
SV	2 days	90 days	196	196	20.44	195	2.31e−50	1.39e−49	***	Self
SV	4 days	30 days	196	196	12.80	195	1.31e−27	7.86e−27	***	Self
SV	4 days	90 days	196	196	18.47	195	1.08e−44	6.48e−44	***	Self
SV	30 days	90 days	196	196	9.55	195	5.54e−18	3.32e−17	***	Self
SV	2 days	4 days	196	196	6.14	195	4.47e−09	2.68e−08	***	Friend
SV	2 days	30 days	196	196	14.28	195	3.97e−32	2.38e−31	***	Friend
SV	2 days	90 days	196	196	19.25	195	5.81e−47	3.49e−46	***	Friend
SV	4 days	30 days	196	196	12.10	195	1.62e−25	9.72e−25	***	Friend
SV	4 days	90 days	196	196	16.98	195	2.77e−40	1.66e−39	***	Friend
SV	30 days	90 days	196	196	9.72	195	1.84e−18	1.10e−17	***	Friend
SV	2 days	4 days	196	196	5.51	195	1.15e−07	6.90e−07	***	Other
SV	2 days	30 days	196	196	11.86	195	8.67e−25	5.20e−24	***	Other
SV	2 days	90 days	196	196	13.85	195	7.90e−31	4.74e−30	***	Other
SV	4 days	30 days	196	196	9.85	195	7.59e−19	4.55e−18	***	Other
SV	4 days	90 days	196	196	12.44	195	1.60e−26	9.60e−26	***	Other
SV	30 days	90 days	196	196	5.43	195	1.66e−07	9.96e−07	***	Other

*Note*. Multiple comparisons corrections are applied per delay-beneficiary.

**Table B.5:** Pairwise comparisons per delay for each delay-beneficiary

y	group1	group2	n1	n2	statistic	df	p	p.adj	signif	delay
SV	Friend	Self	196	196	−10.91	195	5.94e−22	1.78e−21	***	2 days
SV	Friend	Other	196	196	9.99	195	3.03e−19	9.09e−19	***	2 days
SV	Self	Other	196	196	16.48	195	8.36e−39	2.51e−38	***	2 days
SV	Friend	Self	196	196	−9.09	195	1.16e−16	3.48e−16	***	4 days
SV	Friend	Other	196	196	9.58	195	4.57e−18	1.37e−17	***	4 days
SV	Self	Other	196	196	15.85	195	6.88e−37	2.06e−36	***	4 days
SV	Friend	Self	196	196	−5.93	195	1.35e−08	4.05e−08	***	30 days
SV	Friend	Other	196	196	6.81	195	1.15e−10	3.45e−10	***	30 days
SV	Self	Other	196	196	10.14	195	1.12e−19	3.36e−19	***	30 days
SV	Friend	Self	196	196	−6.55	195	5.01e−10	1.50e−09	***	90 days
SV	Friend	Other	196	196	2.35	195	2.00e−02	5.90e−02	ns	90 days
SV	Self	Other	196	196	6.73	195	1.83e−10	5.49e−10	***	90 days

*Note*. Multiple comparisons corrections are applied per delay.

**Table B.6:** Results of the linear mixed effects model of normative subjective values by immediate reward and delay-beneficiary

	DF	DF.res	F	p	p<.05	ges
Delay-beneficiary	2	2151	279.96	< 2.2e−16	***	0.21
ImmReward	1	2151	1908.77	< 2.2e−16	***	0.47
Delay-beneficiary:ImmReward	2	2151	29.73	1.832e−13	***	0.03

**Table B.7:** Pairwise comparisons per delay-beneficiary for each immediate reward

y	group1	group2	n1	n2	statistic	df	p	p.adj	signif	condition
SV	2	4	196	196	7.97	195	1.31e−13	7.86e−13	***	Self
SV	2	6	196	196	13.60	195	4.75e−30	2.85e−29	***	Self
SV	2	8	196	196	19.68	195	3.21e−48	1.93e−47	***	Self
SV	4	6	196	196	10.84	195	9.60e−22	5.76e−21	***	Self
SV	4	8	196	196	17.14	195	8.76–41	5.26e−40	***	Self
SV	6	8	196	196	11.97	195	4.03e−25	2.42e−24	***	Self
SV	2	4	196	196	9.84	195	8.35e−19	5.01e−18	***	Friend
SV	2	6	196	196	16.37	195	1.84e−38	1.10e−37	***	Friend
SV	2	8	196	196	25.28	195	1.93e−63	1.16e−62	***	Friend
SV	4	6	196	196	11.17	195	1.06e−22	6.36e−22	***	Friend
SV	4	8	196	196	19.34	195	3.16e−47	1.90e−46	***	Friend
SV	6	8	196	196	11.62	195	4.78e−24	2.87e−23	***	Friend
SV	2	4	196	196	9.98	195	3.20e−19	1.92e−18	***	Other
SV	2	6	196	196	16.92	195	4.08e−40	2.45e−39	***	Other
SV	2	8	196	196	23.29	195	3.23e−58	1.94e−57	***	Other
SV	4	6	196	196	11.16	195	1.10e−22	6.60e−22	***	Other
SV	4	8	196	196	16.10	195	1.19e−37	7.14e−37	***	Other
SV	6	8	196	196	9.30	195	2.91e−17	1.75e−16	***	Other

*Note*. Multiple comparisons corrections are applied per condition.

**Table B.8:** Pairwise comparisons per immediate reward for each condition

y	group1	group2	n1	n2	statistic	df	p	p.adj	signif	reward
SV	Friend	Self	196	196	−2.80	195	6.00e−03	1.70e−02	*	2
SV	Friend	Other	196	196	5.38	195	2.16e−07	6.48e−07	***	2
SV	Self	Other	196	196	6.39	195	1.21e−09	3.63e−09	***	2
SV	Friend	Self	196	196	−5.72	195	4.05e−08	1.22e−07	***	4
SV	Friend	Other	196	196	7.58	195	1.34e−12	4.02e−12	***	4
SV	Self	Other	196	196	10.54	195	7.73e−21	2.32e−20	***	4
SV	Friend	Self	196	196	−8.55	195	3.53e−15	1.06e−14	***	6
SV	Friend	Other	196	196	7.95	195	1.46e−13	4.38e−13	***	6
SV	Self	Other	196	196	14.27	195	4.15e−32	1.25e−31	***	6
SV	Friend	Self	196	196	−9.89	195	6.01e−19	1.80e−18	***	8
SV	Friend	Other	196	196	6.07	195	6.60e−09	1.98e−08	***	8
SV	Self	Other	196	196	13.66	195	2.96e−30	8.88e−30	***	8

*Note*. Multiple comparisons corrections are applied per immediate reward.

**Figure:**
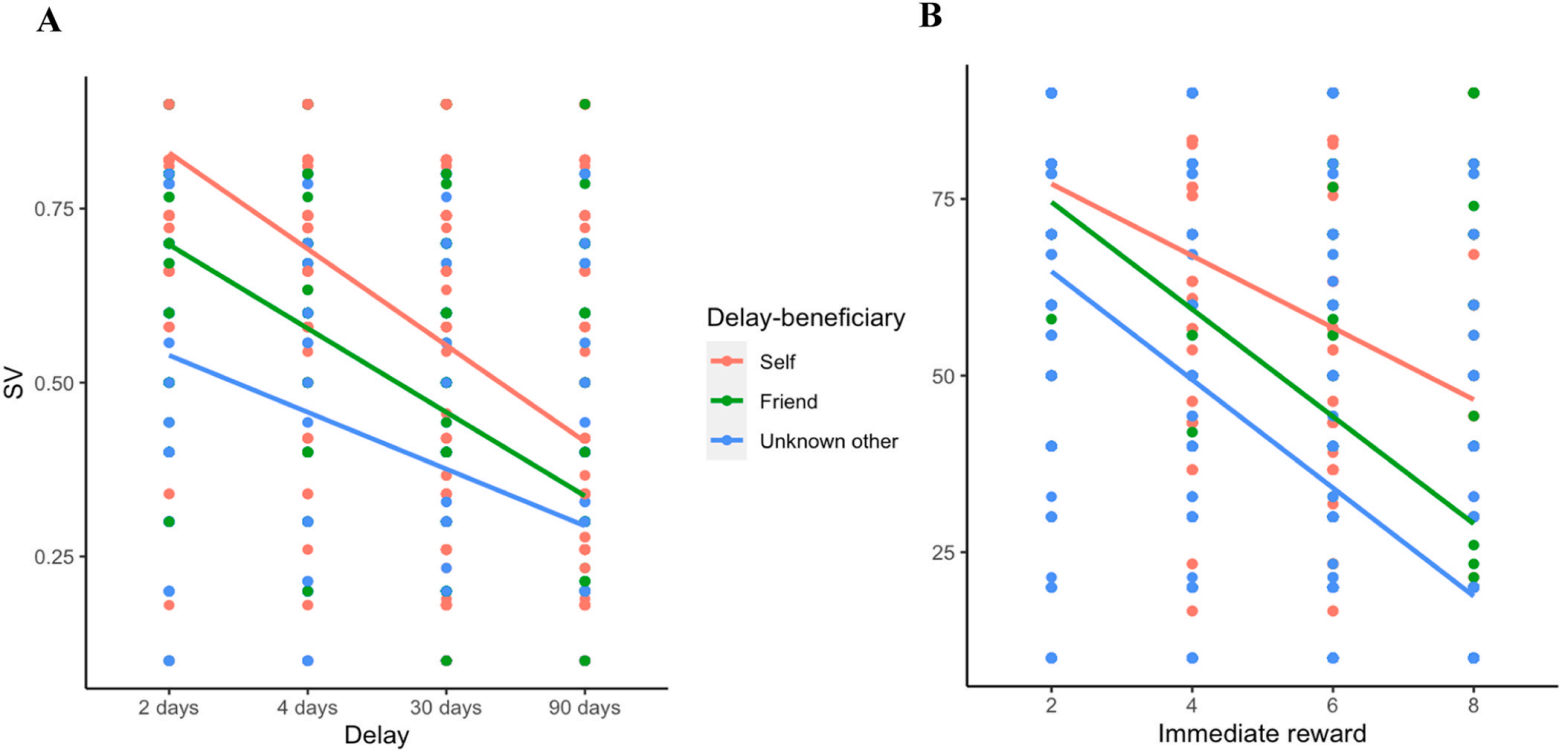
**Figure B.3** Interaction effect of delay beneficiary and delay magnitude (A), and delay-beneficiary with immediate reward (B).

**Table B.9:** Results of the linear mixed effects model of area under the curve value by delay-beneficiary

	DF	DF.res	F	p	p<.05	ges
Delay-beneficiary	2	390	104.69	< 2.2e−16	***	0.35

**Table B.10:** Pairwise comparisons of delay-beneficiary for AUC

y	group1	group2	n1	n2	statistic	df	p	p.adj	signif
AUC	Friend	Self	196	196	−8.74	195	1.05e−15	3.15e−15	***
AUC	Friend	Other	196	196	7.62	195	1.06e−12	3.18e−12	***
AUC	Self	Other	196	196	12.06	195	2.26e−25	6.78e−25	***

**Table B.11:** Results of the linear mixed effects model of area under the curve value by delay-beneficiary and age

	DF	DF.res	F	p	p<.05	ges
Delay-beneficiary	2	390	104.69	< 2.2e−16	***	0.35
Age	1	97.94	4.04	.04718	*	0.04

**Table B.12:** Results of the linear mixed effects model of age for the delay-beneficiary self

	DF	DF.res	F	p	p<.05	ges
Delay-beneficiary	1	97.95	4.44	.03763	*	0.04

**Table B.13:** Results of the linear mixed effects model of age for the delay-beneficiary friend

	DF	DF.res	F	p	p<.05	ges
Delay-beneficiary	1	97.92	2.41	.1239	n.s.	0.02

**Table B.14:** Results of the linear mixed effects model of age for the delay-beneficiary unknown other

	DF	DF.res	F	p	p<.05	ges
Delay-beneficiary	1	97.83	2.79	.09794	n.s.	0.03

**Table B.15:** Bayesian ANOVA of zygosity on overall AUC

Models	P(M)	P(M|data)	BF_M_	BF_10_	error %
Null model	0.50	0.824	4.693	1.00	
Zygosity	0.50	0.176	0.213	0.213	0.05

**Table B.16:** Bayesian ANOVA of zygosity on AUC for the self

Models	P(M)	P(M|data)	BF_M_	BF_10_	error %
Null model	0.50	0.745	2.919	1.00	
Zygosity	0.50	0.255	0.343	0.343	0.04

**Table B.17:** Bayesian ANOVA of zygosity on AUC for the friend

Models	P(M)	P(M|data)	BF_M_	BF_10_	error %
Null model	0.50	0.852	5.747	1.00	
Zygosity	0.50	0.148	0.174	0.174	0.06

**Table B.18:** Bayesian ANOVA of zygosity on AUC for the unknown other

Models	P(M)	P(M|data)	BF_M_	BF_10_	error %
Null model	0.50	0.864	6.368	1.00	
Zygosity	0.50	0.136	0.157	0.157	0.07

**Figure:**
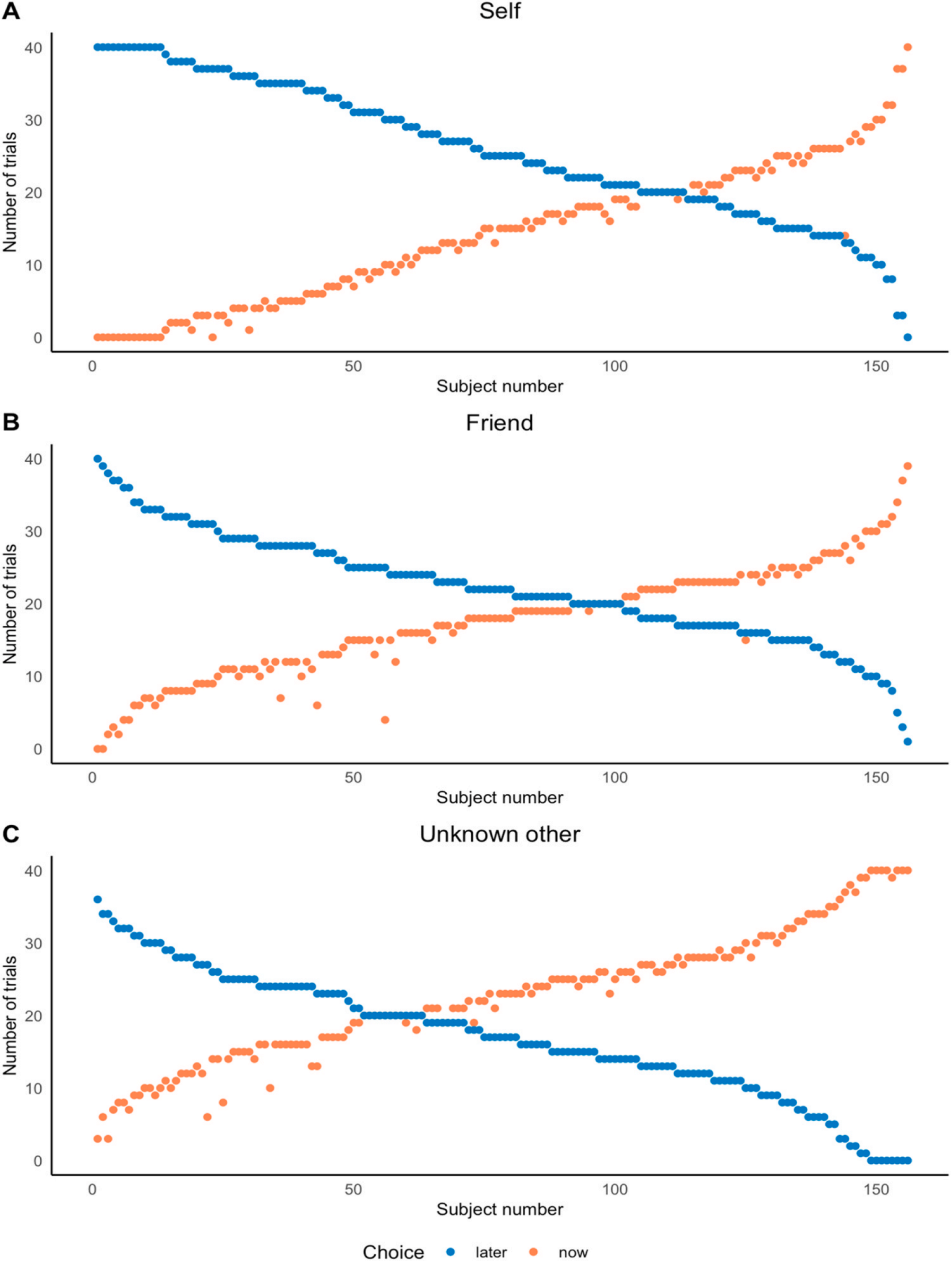
**Figure C.1** The number of trials for the choices later and now by condition.

**Figure:**
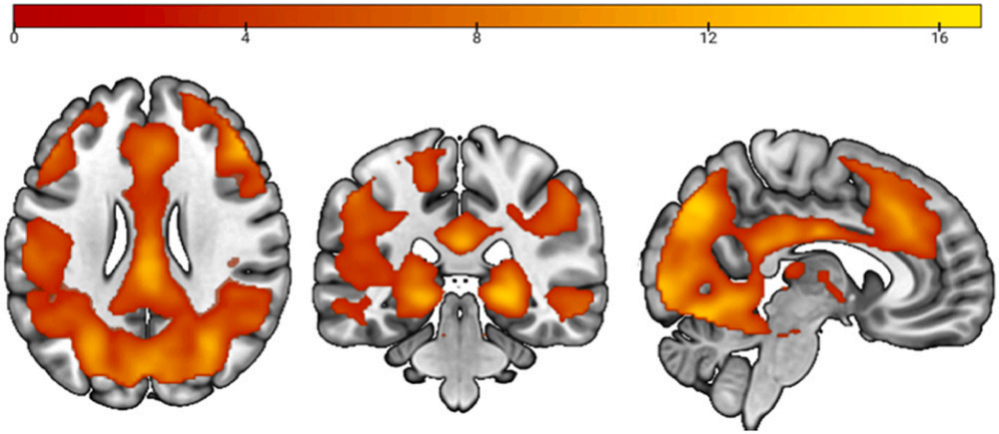
**Figure C.2** All choice trials vs. control. *Note.* FWE and FDR cluster corrected (k = 63631) with uncorrected p-value (p =.001).

**Table C.1:** Paired sample t-test for the main effect choice in the amygdala

Measure 1	Measure 2	t	df	p	BF10
Delayed	Immediate	4.46	137	1.692×10^−5^	859.774

**Table C.2:** Paired sample t-test including bayes factors for the main effect of target in the precuneus, TPJ, and DMPFC

**Measure 1**	**Measure 2**	**t**	**df**	**p**	**BF10**
Precuneus_self	Precuneus_friend	−5.530	137	1.568×10^−7^	72626.978
Precuneus_friend	Precuneus_other	1.364	137	0.175	0.234
Precuneus_self	Precuneus_other	−3.601	137	4.423×10^−4^	41.24
TPJ_self	TPJ_friend	−5.094	137	1.137×10^−6^	11018.764
TPJ_friend	TPJ_other	0.175	137	0.861	0.096
TPJ_self	TPJ_other	−4.437	137	1.858×10^−5^	787.259
DMPFC_self	DMPFC_friend	−5.997	137	1.692×10^−8^	611269.707
DMPFC_friend	DMPFC_other	1.862	137	0.065	0.508
DMPFC_self	DMPFC_other	−3.403	137	8.745×10^−4^	22.078

**Figure:**
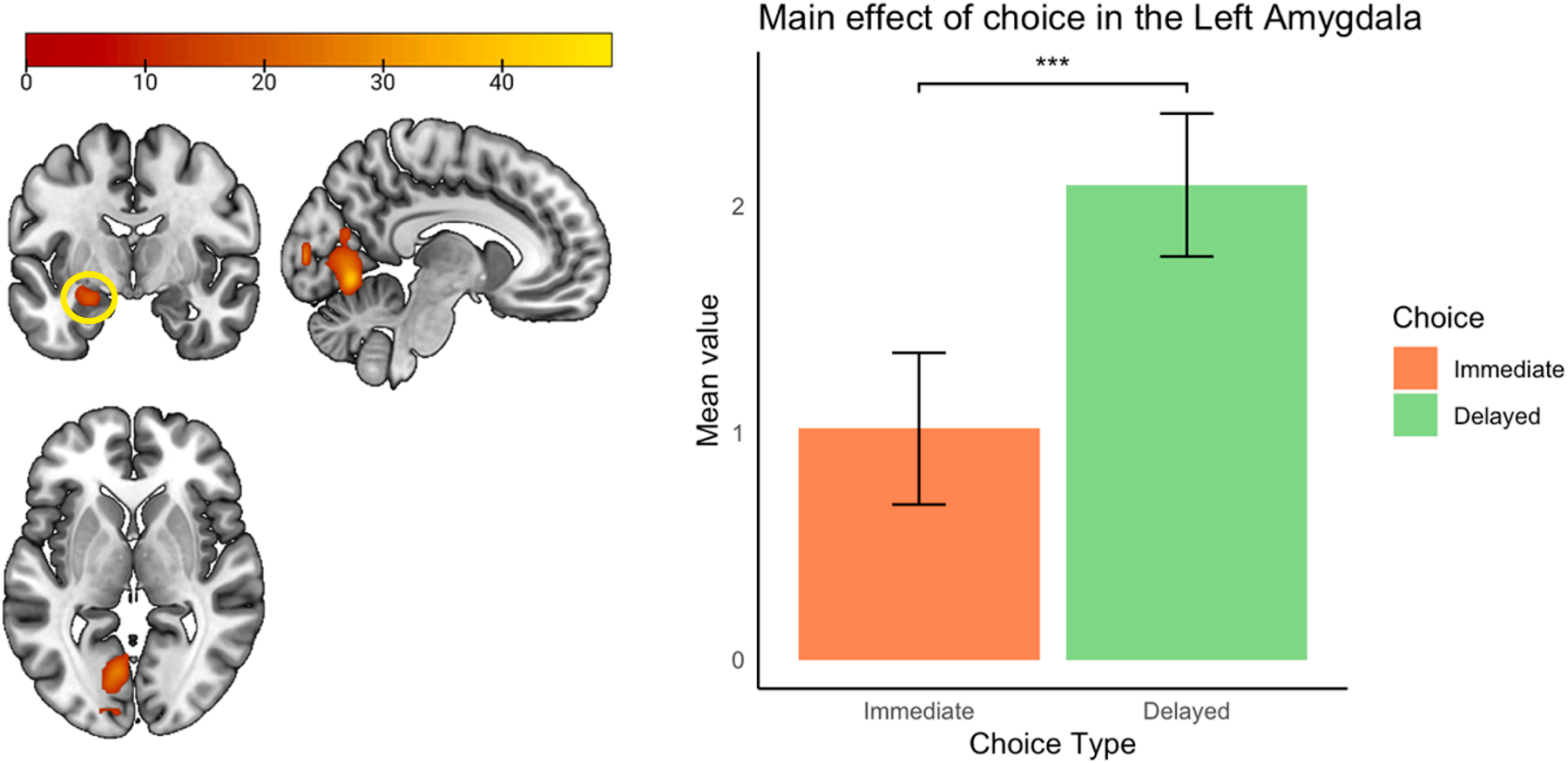
**Figure C.3** Visualization of the main effect of choice in the left amygdala. *Note.* FWE and FDR cluster corrected (k = 123) with initial uncorrected p-value (p =.001).

**Table C.3:** Paired sample t-tests including bayes factors for the interaction effect of delay-beneficiary by choice

**ROI**	**Test**	**t**	**DF**	**p**	**signif**	**BF10**
**ACC**	Self now - later	4,31	137	3,0546E−05	***	493.958
	Friend now - later	0,78	137	0,43609266	NS	0.128
	Other now - later	−3,58	137	0,00047735	***	38.448
**Right TPJ**	Self now - later	3,32	137	0,0011621	**	17.037
	Friend now - later	−0,06	137	0,95018644	NS	0.095
	Other now - later	−3,42	137	0,00083798	***	22.957
**Right Insula**	Self now - later	3,06	137	0,00269062	**	7.967
	Friend now - later	0,83	137	0,40926386	NS	0.132
	Other now - later	−3,98	137	0,00011094	***	148.203
**RdlPFC**	Self now - later	3,24	137	0,00148436	**	13.639
	Friend now - later	0,16	137	0,87087291	NS	0.096
	Other now - later	−3,15	137	0,00199437	**	10.438
**Left TPJ**	Self now - later	2,27	137	0,02478489	*	1.130
	Friend now - later	−1,21	137	0,22749537	NS	0.194
	Other now - later	−3,84	137	0,00018628	***	91.617
**Left Insula**	Self now - later	2,72	137	0,00731404	**	3.265
	Friend now - later	1,03	137	0,30503304	NS	0.159
	Other now - later	−4,09	137	7,254E−05	***	220.109
**Posterior Cingulum**	Self now - later	3,4	137	0,00088783	***	21.777
	Friend now - later	−1,55	137	0,12228182	NS	0.307
	Other now – later	−1,73	137	0,08609012	NS	0.404
**Post Cingulate Cortex**	Self now - later	3,47	137	0,00070675	***	26.826
	Friend now - later	−0,64	137	0,52518002	NS	0.116
	Other now - later	−1,55	137	0,12252622	NS	0.306
**Left temporal cortex**	Self now - later	2,41	137	0,01741325	*	1.530
	Friend now - later	−2,04	137	0,0434194	*	0.705
	Other now - later	−3,11	137	0,00228123	**	9.245

**Figure:**
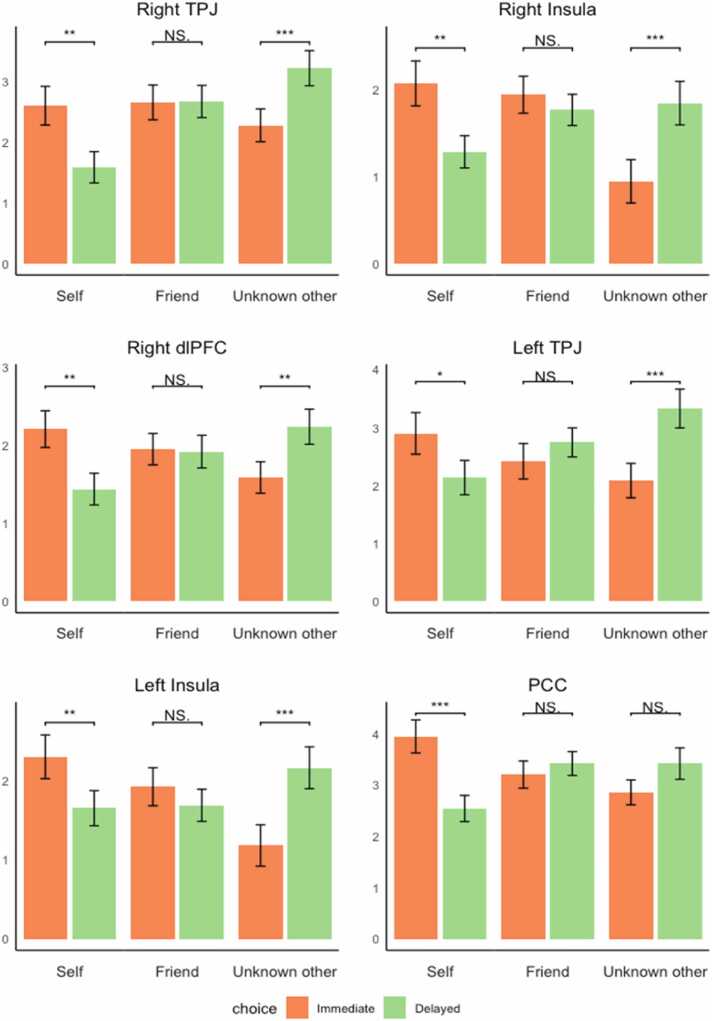
**Figure C.4** Interaction of delay-beneficiary by choice in additional regions

**Figure:**
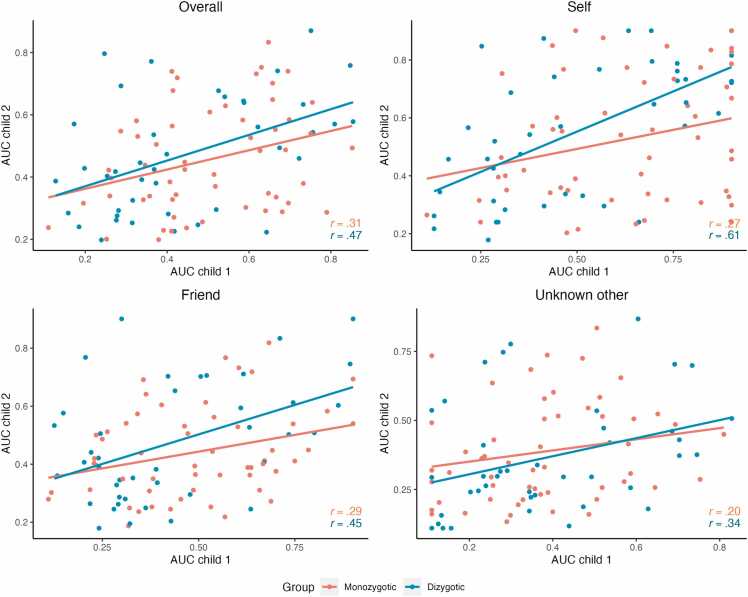
**Figure C.5***Within twin Pearson correlations for AUC values*

## Data Availability

For the LCID consortium, all data will be made available upon request (see https://www.developmentmatters.nl/data-access/ for more information). For the upcoming GUTS consortium in which the social delay discounting task will be used, all data will be made available upon request through a metadata explorer.

## Appendix A

$$\mathrm{normalized SV}=\left(\frac{\mathrm{The number of delayed reward choices at the delay}}{\mathrm{The total number of choices at the delay}}\;x\;\mathrm{the range of plausible SVs}+\mathrm{the lowest possible sv}\right)/10$$

$$\mathrm{AUC trapezoid}=\left(x_{2}-x_{1}\right)\left[{(y}_{1}+y_{2}\right)/\;2]\;$$

*Note.* The calculations used for the normalized SV according to de Water et al. (2023) and AUC according to Myerson et al. (2000). For the AUC calculation, x1 and x2 represents successive delays and y1 and y2 are the subjective values associated with these delays. The calculation can be repeated for each trapezoid, i.e., each delay, after which the sum is equal to the AUC.

## Appendix C

### All choices versus control

We first examined which brain regions are involved in the delay discounting task in general by assessing brain activity for all choices (‘Self – Immediate’, ‘Self – Delay’, ‘Self/Friend - Immediate’, ‘Self/Friend - Delay’, ‘Self/Other - Immediate’, ‘Self/Other - Delay’) versus control using a paired t-test. We excluded participants who had empty cells in any of the choices (i.e., did not make a certain choice, N = 18), resulting in a sample of 138 adolescents (50,7 % females, M_age_ = 15.80, SD_age_= 0.78, age range = 14.27 – 17.14). The contrast (FWE and FDR cluster corrected with an initial uncorrected p =.001, k > 63631) showed increased activity in a wide range of brain regions including prefrontal, parietal, left motor, and subcortical brain regions, see Supplementary Figure 3 and Neurovault link (http://neurovault.org/collections/TARDMHYT/images/807521/).
